# Self-assembly-induced luminescence of Eu^3+^-complexes and application in bioimaging

**DOI:** 10.1093/nsr/nwab016

**Published:** 2021-01-30

**Authors:** Ping-Ru Su, Tao Wang, Pan-Pan Zhou, Xiao-Xi Yang, Xiao-Xia Feng, Mei-Na Zhang, Li-Juan Liang, Yu Tang, Chun-Hua Yan

**Affiliations:** State Key Laboratory of Applied Organic Chemistry, Key Laboratory of Nonferrous Metal Chemistry and Resources Utilization of Gansu Province, College of Chemistry and Chemical Engineering, Lanzhou University, Lanzhou 730000, China; State Key Laboratory of Applied Organic Chemistry, Key Laboratory of Nonferrous Metal Chemistry and Resources Utilization of Gansu Province, College of Chemistry and Chemical Engineering, Lanzhou University, Lanzhou 730000, China; State Key Laboratory of Applied Organic Chemistry, Key Laboratory of Nonferrous Metal Chemistry and Resources Utilization of Gansu Province, College of Chemistry and Chemical Engineering, Lanzhou University, Lanzhou 730000, China; State Key Laboratory of Applied Organic Chemistry, Key Laboratory of Nonferrous Metal Chemistry and Resources Utilization of Gansu Province, College of Chemistry and Chemical Engineering, Lanzhou University, Lanzhou 730000, China; State Key Laboratory of Applied Organic Chemistry, Key Laboratory of Nonferrous Metal Chemistry and Resources Utilization of Gansu Province, College of Chemistry and Chemical Engineering, Lanzhou University, Lanzhou 730000, China; State Key Laboratory of Applied Organic Chemistry, Key Laboratory of Nonferrous Metal Chemistry and Resources Utilization of Gansu Province, College of Chemistry and Chemical Engineering, Lanzhou University, Lanzhou 730000, China; State Key Laboratory of Applied Organic Chemistry, Key Laboratory of Nonferrous Metal Chemistry and Resources Utilization of Gansu Province, College of Chemistry and Chemical Engineering, Lanzhou University, Lanzhou 730000, China; State Key Laboratory of Applied Organic Chemistry, Key Laboratory of Nonferrous Metal Chemistry and Resources Utilization of Gansu Province, College of Chemistry and Chemical Engineering, Lanzhou University, Lanzhou 730000, China; State Key Laboratory of Baiyunobo Rare Earth Resource Researches and Comprehensive Utilization, Baotou Research Institute of Rare Earths, Baotou 014030, China; State Key Laboratory of Applied Organic Chemistry, Key Laboratory of Nonferrous Metal Chemistry and Resources Utilization of Gansu Province, College of Chemistry and Chemical Engineering, Lanzhou University, Lanzhou 730000, China

**Keywords:** rare earth complexes, self-assembly-induced luminescence, bioimaging, temperature and HClO sensing

## Abstract

Design and engineering of highly efficient emitting materials with assembly-induced luminescence, such as room-temperature phosphorescence (RTP) and aggregation-induced emission (AIE), have stimulated extensive efforts. Here, we propose a new strategy to obtain size-controlled Eu^3+^-complex nanoparticles (Eu-NPs) with self-assembly-induced luminescence (SAIL) characteristics without encapsulation or hybridization. Compared with previous RTP or AIE materials, the SAIL phenomena of increased luminescence intensity and lifetime in aqueous solution for the proposed Eu-NPs are due to the combined effect of self-assembly in confining the molecular motion and shielding the water quenching. As proof of concept, we also show that this system can be further applied in bioimaging, temperature measurement and HClO sensing. The SAIL activity of the rare-earth (RE) system proposed here offers a further step forward on the roadmap for the development of RE light conversion systems and their integration in bioimaging and therapy applications.

## Teaser

In this work, a new strategy was proposed to obtain size-controlled Eu^3+^-complex nanoparticles (Eu-NPs) with self-assembly induced luminescence (SAIL) characteristics without encapsulation or hybridization. And the SAIL activity of the rare-earth (RE) system proposed here offers a further step forward on the roadmap for the development of RE light conversion and their integration in bioimaging and therapy applications.

## INTRODUCTION

The unique properties of rare-earth (RE) complexes including ligand-sensitized energy transfer, fingerprint-like emissions and long-lived emissions [1–3], make them promising materials for many applications such as LED devices [4,5], optical encoding [6,7], luminescence imaging/detection [8–11] and time-resolved luminescence detection [12,13]. In particular, the use of RE luminescent materials for *in vitro* and *in vivo* imaging can easily eliminate the autofluorescence of organisms and any interference from background fluorescence [14]. However, most RE complexes have poor solubility and stability in aqueous solution and their luminescence can be easily quenched by nearby X−H (X = O, N, C) oscillators [15–17], which limits their further application in aqueous solutions and bioimaging. Consequently, improving their luminescence performance as well as dispersibility has become a key issue in expanding the application of RE complexes. Until now, extensive efforts have been devoted to increasing the luminescence intensity of RE complexes, such as increasing structural rigidity, adjusting coordination numbers, replacing ligand C–H bonds with C–F bonds and changing the electron-donating or electron-withdrawing characteristics of substituents [18–20].

Recently, assembly-induced emission materials, such as room-temperature phosphorescence (RTP) materials and aggregation-induced emission (AIE) luminogens (AIEgens), have become research hotspots [21–24]. Ma *et al.* summarized the recent advances in assembly-induced emission of amorphous RTP materials [25]. Tang *et al.* summarized the progress of AIE and its related fields, and suggested its application prospects in materials and biological sciences [26]. Compared to these emitting materials, RE complexes have a relatively complicated sensitized luminescence mechanism. In different sensitization processes, the energy transfer from the excited triplet state of the ligands to the excited state of the RE ions (T_1_-RE^*^) is the main cause of sensitization [27]. Therefore, increasing the possibility of intersystem crossing to the ligand triplet excited state (S_1_-T_1_) and reducing the non-radiative decay would be beneficial to the luminescence of RE complexes.

Recent studies have shown that supramolecular assembly can build highly water-dispersible nanostructures through non-covalent intermolecular force, which would allow the RE complex to be applied in more areas [28–30]. Kimizuka *et al.* encapsulated Eu^3+^-complexes into amphiphilic matrices to reduce the quenching of water molecules and increase the stability of complexes by supramolecular assembly [31]. With an organic–inorganic assembling strategy, Li *et al.* also used RE complexes as emitting sources to realize robust luminescent hydrogels [32]. However, it is difficult to predict the assembly and to control the particle size distribution by simply dispersing RE complexes into host matrices. As known, self-assembly driven by intermolecular forces, such as hydrophobic–hydrophobic, hydrogen bonding and aromatic π–π stacking, has a high degree of orientation and predictability, and is a powerful strategy for synthesizing nanostructures with precise sizes and shapes [33]. At the same time, such intermolecular interaction forces can change the intermolecular distance, limit the rotation of the ligand molecules and regulate the energy transfer from the ligands to the central RE ions [34].

**Scheme 1.**
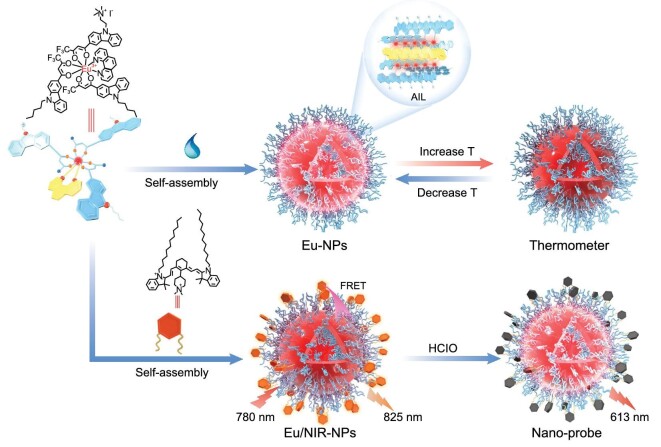
Schematic illustration of the synthetic protocol of the self-assembly-induced luminescence (SAIL) of Eu-complex and its application for the bioimaging of temperature and HClO.

Here, a new strategy was proposed to obtain size-controlled Eu^3+^-complex nanoparticles (Eu-NPs) with self-assembly-induced luminescence (SAIL) characteristics without encapsulation or hybridization. The amphiphilic Eu^3+^-complex (Eu(THB)(THA)_2_Phen) possessing carbazole derivative ligands, with highly π–π conjugated electron structure, could self-assemble into Eu-NPs with excellent water dispersibility and controllable particle size in aqueous solution (Scheme 1). We envisaged that adjusting the molecular polarity of the ligands and transferring the RE complexes from the organic phase to the water phase could cause the RE complexes to assemble into NPs with good water dispersibility. By studying the changes in luminescence lifetimes and quantum yields in aqueous solution, we found that (i) self-assembly could effectively shield the water molecules in the luminescent center and thus reduce the quenching effect of the water molecules from the vibration of the O−H bond; (ii) when the molecules are self-assembled together, they restrain each other, and their movement within the molecules is restricted. This will greatly limit the intramolecular rotation or vibration of Eu^3+^-complexes, thus resulting in the enhancement of luminescence in aqueous conditions. Also, this system could be used for bioimaging application for the detection of temperature and HClO by steady-state fluorescence and time-resolved assay as shown in Scheme 1. We believe that the SAIL activity of the self-assembled RE complexes system proposed here paves a new way for the development of RE light conversion systems and their integration in bioimaging and therapy applications.

## RESULTS AND DISCUSSION

### Self-assembly morphology and properties of Eu^3+^-complexes in aqueous solution

First, we successfully synthesized hydrophilic ligand N,N,N-trimethyl-2-(3-(4,4,4-trifluoro-3-oxobutanoyl)-9H-carbazol-9-yl)ethan-1-aminium (HTHB) (Scheme S1) and hydrophobic ligand 4,4,4-trifluoro-1-(9-pentyl-9H-carbazol-3-yl) butane-1,3-dione (HTHA) (Scheme S2). The synthetic steps and ^1^H and ^13^C NMR spectra are listed in the supporting information. Then, a new amphiphilic Eu^3+^-complex (Eu(THB)(THA)_2_Phen, Scheme S3) was synthesized according to our previous work [35]. The successful synthesis of Eu^3+^-complex was confirmed by high resolution mass spectrometry (HRMS), MALDI-TOF and elemental analysis as shown in the supporting information.

**Figure 1.**
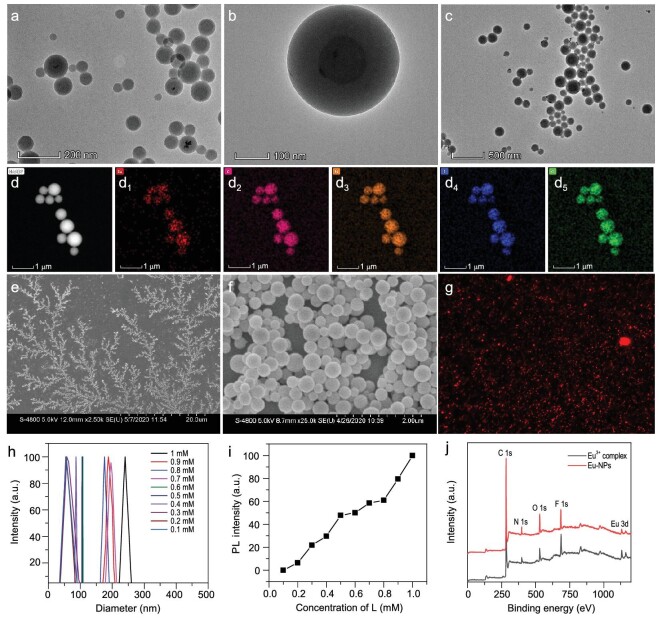
(a and b) TEM images of Eu-NPs at the concentrations of 0.5 mM. (c) TEM images of Eu/NIR-NPs. (d) The elemental mapping images of Eu-NPs. (e and f) SEM images of Eu-NPs at different concentrations of 0.005 and 1 mM. (g) Confocal fluorescence microscope images of Eu-NPs at the concentration of 1 mM (*λ*_ex_ = 405 nm; *λ*_em_ = 600–640 nm). (h) Hydrodynamic particle size distribution of Eu-NPs at different concentrations from 0.1 to 1 mM in aqueous solution measured by DLS. (i) Normalized luminescence intensity of Eu-NPs with different size distributions (the total Eu^3+^-complexes remained the same). (j) XPS spectra of Eu(THB)(THA)_2_Phen and Eu-NPs.

The self-assembly behavior of Eu(THB)(THA)_2_Phen in aqueous solution was studied by dissolving the Eu^3+^-complexes in a small amount of acetone, and then dispersing them into aqueous solution with ultrasound treatment. Transmission electron microscopy (TEM) images showed that the Eu(THB)(THA)_2_Phen complex self-assembled into novel spherical micelle NPs (Eu-NPs) with a double-layer structure (Fig. 1a and b). To expand the application of Eu-NPs in constructing Föster resonance energy transfer (FRET) systems, we also co-assembled Eu(THB)(THA)_2_Phen with amphiphilic near-infrared dye IR-780. The TEM images showed that they could also be co-assembled into nanospheres with uniform morphology and good dispersion (Fig. 1c). The elemental mapping of Eu^3+^-NPs showed that F, O, Eu, N and C elements were evenly distributed on the vesicles (Fig. 1d). The energy dispersive spectroscopy (EDS) spectrum of Eu-NPs further confirmed the existence of these elements (Fig. S1).

To study the influence of Eu(THB)(THA)_2_Phen concentration on the self-assembly of nanoparticles, scanning electron microscopy (SEM) images were collected and particle size distribution of Eu-NPs was measured at different concentrations of Eu(THB)(THA)_2_Phen (0.005 mM, 0.01 mM, 0.02 mM, 0.04 mM, 0.06 mM, 0.8 mM, 0.1 mM, 0.2 mM, 0.3 mM, 0.4 mM, 0.5 mM, 0.6 mM, 0.7 mM, 0.8 mM, 0.9 mM and 1.0 mM). It was found from SEM images (Fig. 1e and f and Fig. S2) that Eu(THB)(THA)_2_Phen assembled more efficiently in aqueous solution with increasing concentration, and the particle size and dispersion of Eu-NPs gradually increased. The confocal microscope images (Fig. 1g) showed that Eu-NPs had good dispersibility and luminescence properties. The results of dynamic light scattering (DLS) analysis (Fig. 1h) showed that the average hydrodynamic diameter of Eu-NPs at different concentrations of Eu(THB)(THA)_2_Phen (from 0.10 mM to 1.0 mM) increased from 56 ± 5 nm to 245 ± 5 nm. Eu(THB)(THA)_2_Phen self-assembled more efficiently in aqueous solution with increasing concentration and its luminescence intensity increased with the increase in its concentration (Fig. 1i). We labeled Eu^3+^-NPs at different concentrations of Eu(THB)(THA)_2_Phen (from 0.10 mM to 1.0 mM) as Eu-NPs-0.1 to Eu-NPs-1.0. By comparing the changes in X-ray photoelectron spectroscopy (XPS) data before and after the self-assembly of Eu(THB)(THA)_2_Phen, it was further verified that the same elements were present before and after self-assembly (Fig. 1j). To assess the universality of the proposed self-assembly strategy, we also synthesized three other Eu^3+^-complexes, including Eu(THA)_3_Phen, Eu(tta)_3_(dpqt) and Eu(THA)_3_(dpqt) (Schemes S5–S8) and studied their self-assembly property. As shown in Fig. S3, they all assembled into NPs with different morphologies in aqueous solution. Furthermore, RE complexes are often coordinated competitively by other molecules, such as PO_4_^3−^, amino acids, etc., which can affect the luminescence. To study whether self-assembly increases the resistance of RE complexes to interference by other molecules, interference experiments were conducted. The results showed that other substances (H_2_PO_4_^−^, NO_2_^−^, HPO_4_^2−^, HCO_3_^−^, CO_3_^2−^, SO_4_^2−^, PO_4_^3−^, HSO_3_^−^, SCN^−^, CN^−^, PBS, GSH, Asp, Arg, Cys) did not interfere noticeably with the fluorescence of the self-assembled nanoparticles Eu-NPs-1.0 (Fig. S4). We also studied the shelf stability of Eu-NPs-1.0 in aqueous solution, and found that it is very stable in aqueous solution (Fig. S5).

In order to further investigate the self-assembly behavior, calculations were carried out using the periodic density functional theory (DFT) method and the local-density approximation (LDA). In the optimized structure of Eu(THB)(THA)_2_Phen, the carbazole ring of the HTHA group was in a plane, while the carbazole ring of HTHB was on the opposite side of HTHA (Fig. S6a). From the optimized structure of molecular stacking, it can be seen that the molecules are stacked in antisymmetric manner. The distance between the mass centers of the carbazole rings of the ligand HTHB was 3.786 Å, which indicated that there was a π–π stacking force in the molecular aggregation (Fig. S6b). Therefore, the non-covalent forces that induce Eu^3+^-complexes to self-assemble into Eu-NPs are hydrophobic–hydrophobic, hydrogen bonding and aromatic π–π stacking forces.

### Photoluminescence and UV-vis spectra of Eu^3+^-complexes in binary solvents of organic and water

**Figure 2.**
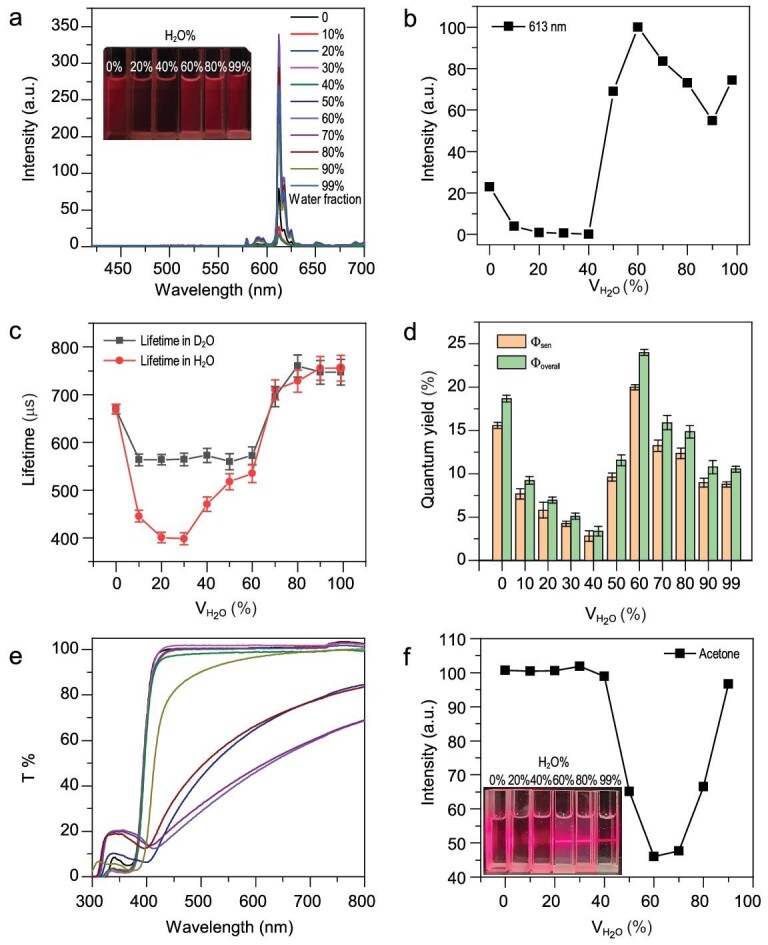
(a) Photoluminescence spectra of Eu(THB)(THA)_2_Phen (50 μM) in acetone/water mixtures (inset: fluorescent photographs of Eu(THB)(THA)_2_Phen at different water fractions). (b) The normalized luminescence intensity of Eu(THB)(THA)_2_Phen (50 μM) in acetone/water mixtures. (c) The lifetime at the wavelength of 613 nm for Eu(THB)(THA)_2_Phen (50 μM) in different acetone/D_2_O (black line) or acetone/H_2_O (red line) mixtures. (d) Luminescence quantum yield (*Φ*_overall_) and the efficiency of energy transfer (*Φ*_sen_) from ligand to Eu^3+^ ions of Eu(THB)(THA)_2_Phen (50 μM) in different acetone/water mixtures. (e and f) Dependence of the optical transmittance at 600 nm of Eu(THB)(THA)_2_Phen in different acetone/water mixtures (inset: photographs of Eu^3+^ complexes at different water fractions and their Tyndall effect).

The luminescence of the Eu^3+^-complexes was studied in mixed solutions of organic solvents (THF, MeCN, ethanol, DMSO, DMF and acetone) and water. We investigated the luminescence changes of the four Eu^3+^-complexes (25 μM) (Eu(THB)(THA)_2_Phen, Eu(THA)_3_Phen, Eu(tta)_3_(dpqt) and Eu(THA)_3_(dpqt)) (Fig. S7a_1_–c_1_) in varying proportions of acetone/water binary mixtures by adding acetone solution to water followed by stirring for 5 min. As shown in Fig. 2a and b and Fig. S7a_2_–c_3_, when V_H2O_ < 40%, the luminescent intensities of Eu^3+^ at emission wavelength of 613 nm (^5^D_0_→^7^F_2_) decreased slightly, which could be attributed to the increased quenching of the luminescence of Eu^3+^ through the excitation of O−H vibrations. Interestingly, when V_H2O_ > 40%, the luminescent intensities sharply increased. On the contrary, with the increase in water content, the ligand fluorescence of complexes Eu(tta)_3_(dpqt) and Eu(THA)_3_(dpqt) at emission wavelength of 460 nm was quenched by the well-known aggregation-caused quenching (ACQ) effect [36]. Besides, when the excitation wavelength and concentration of Eu(THB)(THA)_2_Phen (5.0 μM, 25.0 μM, 50.0 μM, 100.0 μM) were varied, the change in luminescence intensity showed the same phenomenon (Figs S8 and S9). Nevertheless, the luminescence slightly decreased when water fraction exceeded 60%. This was because the particle size became smaller with increasing water content (Fig. S10), which lowered the number of self-assembled Eu^3+^-complexes [29].

We assumed that the enhancement of luminescence was due to the self-assembly of Eu^3+^-complexes as the amount of water increased. Therefore, the luminescent center was likely hidden in the hydrophobic cavity, reducing the influence of the surrounding water on its luminescence. To verify our hypothesis, the number of water molecules in the first coordination sphere of Eu^3+^ ions in Eu(THB)(THA)_2_Phen was estimated by using the following equation (1) [37]

(1) $$\begin
{equation}q\ = {\rm{\ }}1.2\left( {\tau _H^{ - 1} - \tau _D^{ - 1} - 0.25} \right),\end{equation}$$

where *q* represents the number of coordinated water molecules, and *τ*_H_ and *τ*_D_ are the luminescence lifetimes measured in H_2_O and D_2_O, respectively. Using this formula, it was determined that the number of coordinated water molecules q in Eu(THB)(THA)_2_Phen was 0.6 ± 0.1, indicating that almost one water molecule was coordinated with two Eu^3+^ ions in Eu(THB)(THA)_2_Phen. In contrast, when the proportion of water exceeded 60%, the luminescence decay rate of Eu(THB)(THA)_2_Phen in H_2_O was only slightly lower than that in D_2_O, which proved that the number of coordinated water molecules in the first coordination sphere of Eu^3+^ ions in Eu(THB)(THA)_2_Phen was very small (Fig. 2c). However, in the system of D_2_O/acetone, when the water content was greater than 40%, the fluorescence lifetime still showed an increasing trend, which indicated that excluding the quenching effect of water molecules was not the only reason for the enhanced fluorescence. Therefore, it can be argued that the self-assembly leads to physical and spatial limitations, which greatly hinder the intramolecular rotation or vibration of the Eu^3+^-complex, resulting in enhanced luminescence under aqueous conditions.

The quantum yields (*Φ*) of Eu(THB)(THA)_2_Phen were studied in varying proportions of acetone/water binary mixtures. The overall luminescence quantum yield (*Φ*_overall_) followed the same trend as the luminescence intensity (Fig. 2d and Table S1), which increased from (3.4 ± 0.1)% to (24.0 ± 0.1)% when the water fraction increased from (40.0 ± 0.1)% to (60.0 ± 0.1)%. We also studied the efficiency of the energy transfer (*Φ*_sen_) from ligand to Eu^3+^ ions of Eu(THB)(THA)_2_Phen (50 μM) in different acetone/water mixtures. The relationship between *Φ*_overall_ and *Φ*_sen_ can be expressed by the following formula (2) [38],

(2) $$\begin
{equation}{\Phi_{{\rm{overall}}}} = {\Phi_{{\rm{sen}}}}{\Phi_{{\rm{Eu}}}}.
\end{equation}$$

The intrinsic quantum yield of Eu^3+^ (*Φ*_Eu_) was estimated by the following equation (3), and it is assumed that the decay process at 77 K in deuterated solvents is purely radiative [39,40],

(3) $$\begin
{equation}{\Phi _{{\rm{Eu}}}} = {\tau _{{\rm{obs}}}}\left( {298\,{\rm{K}}} \right)/{\tau _{{\rm{obs}}}}\left( {77\,{\rm{K}}}\right)\!.
\end{equation}$$

The changes of *Φ*_sen_ in different acetone/water ratios demonstrated the inherent reason for the changes in fluorescence intensity. The calculation results showed that the change trend of *Φ*_sen_ was consistent with the fluorescence intensity. At the same time, the internal quantum yield increased from (9.6 ± 0.1)% (water 40%) to (20.0 ± 0.1)% (water 60%) (Table S1). This further proved that the assembly greatly limited the intramolecular rotation or vibration of Eu^3+^ complexes, which facilitated energy transfer from the ligand to the central Eu^3+^ ions.

We also studied the change in luminescence or light transmittance of Eu(THB)(THA)_2_Phen in other mixed solutions of organic solvents (THF, MeCN, ethanol, DMSO and DMF) and water. The general trend of luminescence change was the same as that in the acetone/water mixed solvent, but the sharp change in fluorescence or light transmittance occurred at different proportions of water component (Figs S11 and S12). This may be caused by the fact that the solubility of Eu(THB)(THA)_2_Phen differs in different organic solvents/water. By measuring the change in light transmittance at 600 nm in different acetone/water ratios, it was found that the light transmittance decreased significantly when water fraction was greater than 40% (Fig. 2e and f). Turbidity and Tyndall effect were also observed from photographs of Eu^3+^ complexes at water fraction >40% (inset of Fig. 2f). These experimental results provided further evidence for the self-assembly of the Eu^3+^-complexes when the water ratio was greater than 40%. When the proportion of water was greater than 60%, its light transmittance began to increase again. This was consistent with a slight decrease in fluorescence intensity, further confirming that the assembled particles became smaller and the number of effective assemblies was reduced.

### Temperature-sensing performance of Eu-NPs

With the rapid development of scientific research, technology applications and industrial production, traditional thermometers cannot meet the requirements for measurement of temperature in some particular areas, including micron- or nanodevices, biological processes and disease diagnoses [41]. In addition, accurate detection of the temperature distribution of living cells, especially the temperature distribution of cancer cells that have higher temperature than normal tissues due to the increased metabolic activity, could greatly enhance the understanding of their pathology and physiology, thereby optimizing diagnoses and treatment processes (for example, in hyperthermia tumor treatment and photodynamic therapy) [42,43]. Therefore, it is urgent to develop nanoscale temperature measurement devices to achieve temperature measurement of specific lesions. At the same time, controlling the characteristics of nanomaterials (for example, size dispersion, surface modification) can help achieve different spatial resolutions and positioning.

**Figure 3.**
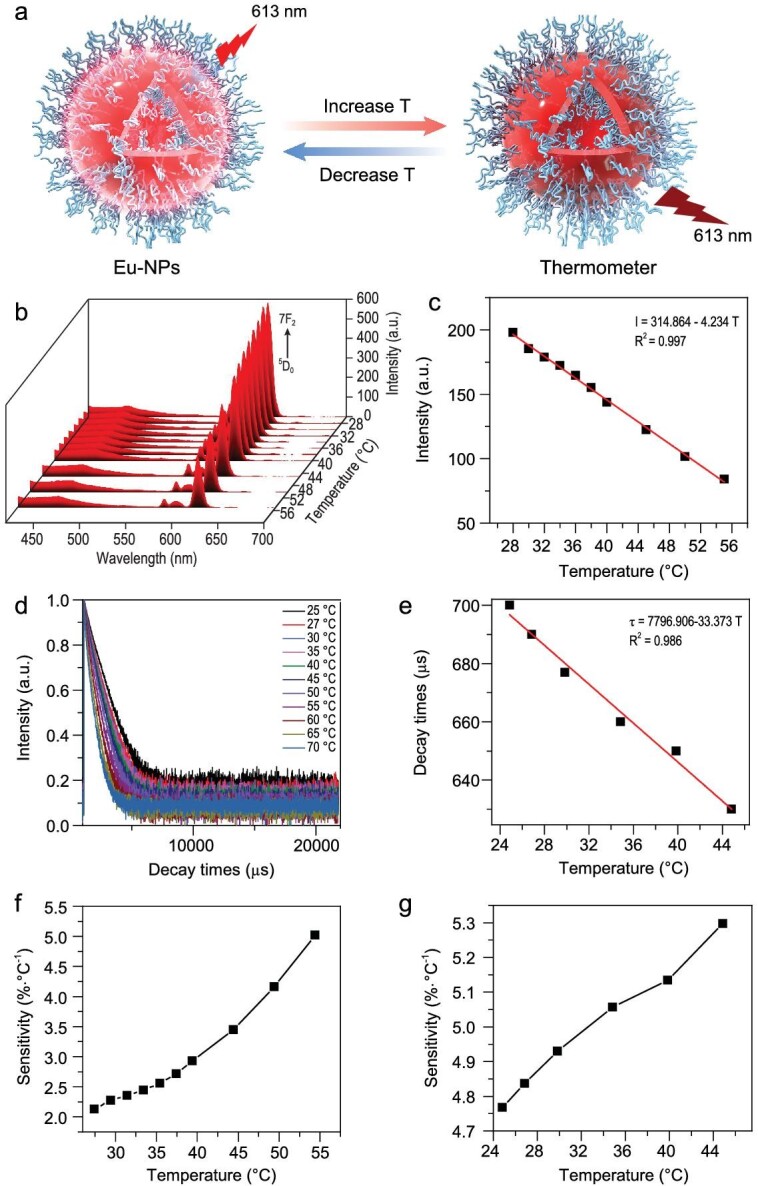
(a) Schematic illustration of Eu-NPs sensing temperature. (b) Photoluminescence spectra of Eu-NPs-0.5 at different temperatures in water media. (c) The linear relationship between the luminescence intensity (*λ*_em_ = 613 nm) and different temperatures in water (from 24°C to 55°C). (d) The decay curves at the wavelength of 613 nm for Eu-NPs-0.5 at different temperatures. (e) The linear relationship between the luminescence decay time (*τ*_613_) and different temperature (from 24°C to 45°C). (f) The relative sensitivity (Sr) for Eu-NPs-0.5. (g) The relative sensitivity (Sr) of temperature-dependent lifetime for Eu-NPs-0.5.

So far, there is no report on the application of self-assembled RE complexes as nanometer thermometers. In order to study the response of the Eu-NPs to temperature changes, we selected Eu-NPs-0.5 as a nanothermometer and measured its steady-state luminescence and transient-state luminescence at different temperatures (Fig. 3a). As shown in Fig. 3b, the luminescence intensity of ^5^D_0_–^7^F_2_ transition (*I*_613_) reduced as the temperature increased. The linear relationship between temperature and *I*_613_ could be fitted as a function of equation (4)

(4) $$\begin
{equation}{I_{613}} = 314.864 - 4.23\,T,\end{equation}$$

with a correlation coefficient (R^2^) of 0.997 (Fig. 3c). Quantitative comparison of thermometers operated by different mechanisms can be performed using relative sensitivity, which is defined as equation (5),

(5) $$\begin
{equation}{\rm{S}} = \left| {\frac{{\partial \left( {{I_{613}}} \right)/\partial \left( T \right)}}{{{I_{613}}}}} \right|.
\end{equation}$$

The fluorescence lifetime value has good stability, and it does not depend on the penetration depth of biological tissue, the concentration of the probe, light scattering, reflection or the intensity fluctuation of the excitation source. Thus, it breaks through the limitations of traditional steady-state fluorescence detection, adding an independent new dimension of information to fluorescence imaging. Compared with traditional organic fluorescent probes, RE complexes as nanoprobes have unique photophysical advantages, including longer emission lifetimes and time-gated and time-resolved measurements. Therefore, we measured the change in luminescence lifetime of ^5^D_0_–^7^F_2_ (613 nm) induced by the change in temperature from 25°C to 70°C to evaluate the potential application of Eu-NPs-0.5 as a lifetime thermal sensor. Figure 3d shows the luminescence decay curve of Eu-NPs-0.5 from 25°C to 70°C, which indicates that the luminescence lifetime of ^5^D_0_–^7^F_2_ (613 nm) decreased with increasing temperature. The linear relationship between the luminescence decay time (*τ*_613_) and temperature indicated that the luminescence lifetime and temperature (from 24°C to 45°C) had a good linear relationship with the correlation coefficient (R^2^) of 0.986 (Fig. 3e). The linear relationship between temperature and luminescence decay time (*τ*_613_) could be fitted as a function of the following equation (6),

(6) $$\begin
{equation}{\tau _{613}} = 7796.906-33.373T.
\end{equation}$$

Figure 3f revealed that the maximum relative sensitivity and the minimum relative sensitivity were 5.1%·°C^−1^ and 2.3%·°C^−1^ at 55.0°C and 25°C, respectively. It indicated that Eu-NPs-0.5 can be applied as a thermal sensor to situations that require precise temperature measurement.

As shown in Fig. 3g, the maximum relative sensitivity and the minimum relative sensitivity were 5.3%·°C^−1^ and 4.75%·°C^−1^ at 45.0°C and 25°C, respectively. It indicated that Eu-NPs-0.5 can be applied as a thermal sensor to situations that require precise temperature measurement. The maximum relative sensitivity of Eu-NPs was higher than that of other reported RE thermal probes (Table S2).

For fluorescent probes used as temperature sensors, thermal stability is also critical. The thermal stability of Eu-NPs-0.5 has been studied by TGA-DSC technology. Figure S13 shows that the melting point and decomposition temperature were 208°C and 337°C, respectively. Thus, Eu-NPs-0.5 has good thermal stability and can be used as a temperature sensor. At the same time, we also studied the temperature measurement properties of high and low temperature cycles, and found that Eu-NPs-0.5 has good cycling properties for temperature measurement (Fig. S14). We studied the particle size distribution of the Eu-NPs-0.5 at different temperatures (from 25°C to 55°C), and found that the particle size did not change significantly with the temperature change, which showed that the temperature change within the measured temperature range cannot destroy the assembly (Fig. S15).

### Application in sensing of HClO

**Figure 4.**
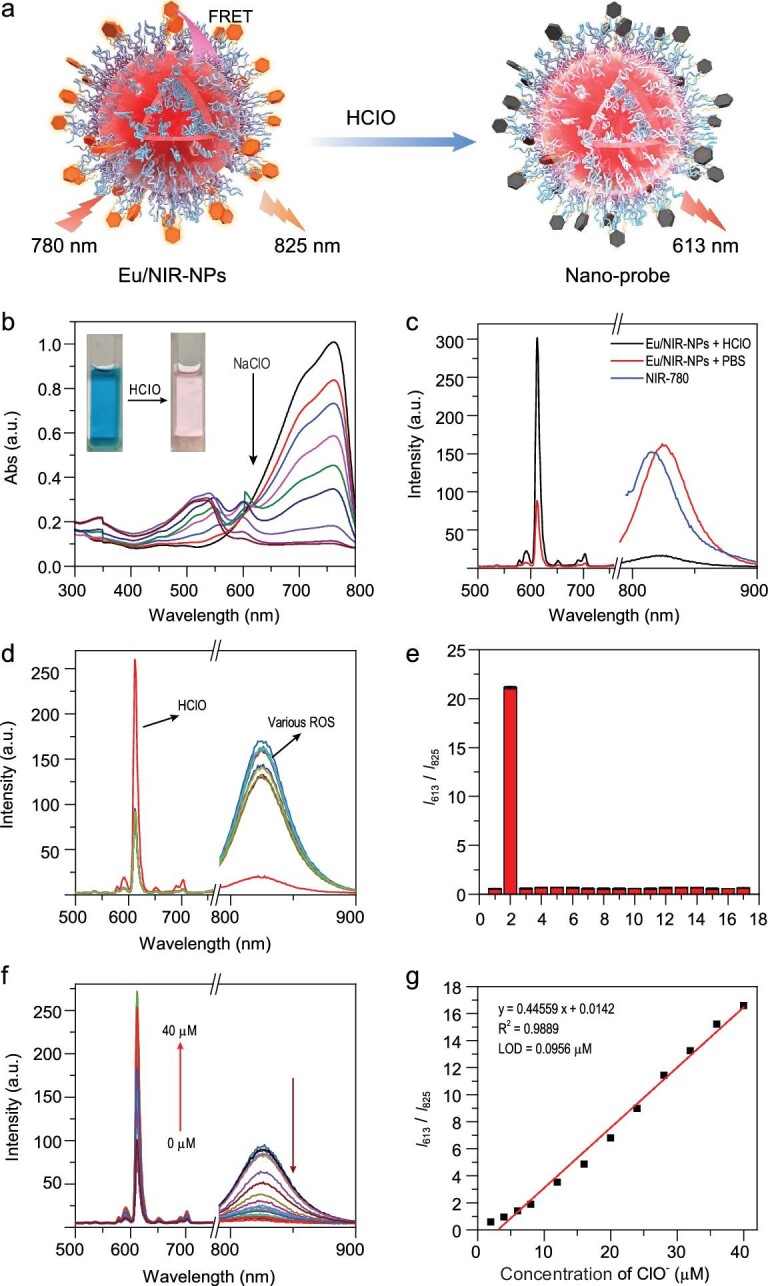
(a) Schematic illustration of Eu/NIR-NPs detecting HClO. (b) The UV-vis spectra of Eu/NIR-NPs in different concentrations of HClO in water media. (Inset: photographs of Eu/NIR-NPs solution before or after adding NaClO.) (c) The fluorescence spectra of Eu/NIR-NPs, IR-780 and Eu/NIR-NPs with NaClO. (d) Fluorescence spectra of Eu/NIR-NPs (1.0 mg/mL) after adding 40.0 μM of various ROS and NaClO in HEPES buffer solution (10 mM, pH 7.4). (e) The competing responses of Eu/NIR-NPs to various substances and NaClO in HEPES buffer solution (10 mM, pH = 7.4). (1) PBS, (2) NaClO, (3) $ \cdot $OH, (4) ^1^O_2_, (5) NO, (6) ONOO^−^, (7) t-BuOOH, (8) H_2_O_2_, (9) GSH, (10) Cys, (11) Glu, (12) PO_4_^3−^, (13) CO_3_^2−^, (14) HS^−^, (15) Cl^−^, (16) SO_4_^2−^, (17) NO_2_^−^, (18) NO_3_^−^. *λ*_ex_ = 780 nm. (f) Fluorescence response of Eu/NIR-NPs (1.0 mg/mL) towards NaClO (0–40.0 μM). (g) The linear relationship between the fluorescence ratio (*I*_613_/*I*_825_) and different NaClO concentrations (from 0 to 40.0 μM). *λ*_ex_ = 780 nm.

Hypochlorous acid (HClO) is produced by hydrogen peroxide and chloride ions under the catalytic action of myeloperoxidase (MPO) in the body. Growing attention is given to the identification and detection of HClO, due to its vital role in bioassay of physiological processes, such as cell differentiation, migration, conduction and immunity, etc. [44]. The FRET-based fluorescent probes have the advantages of a large Stokes shift, ratiometric sensing, dual/multi-analyte responsive systems, etc., which have gained strong research interest [45]. At the same time, most near-infrared fluorescent dyes are prone to fluorescent photobleaching, and most HClO fluorescent probes are Turn-Off type, which is not conducive to accurate and sensitive detection. Here, based on the FRET system constructed by HClO fluorescent probe IR-780 and Eu^3+^ complexes, ratiometric HClO luminescence nanoprobe Eu/NIR-NPs was constructed by co-assembly, in which two-photon-sensitized amphiphilic Eu(THB)(THA)_2_Phen directly co-assembled with cationic IR-780 (Fig. 4a). Based on the good overlap between the emission peaks of Eu(THB)(THA)_2_Phen and the strong absorption of IR-780, (Eu/NIR-NPs) and Eu(THB)(THA)_2_Phen were used as energy donors in this assembly, and IR-780 was used as energy acceptor (Figs S16 and S17). First, we studied the assembly and fluorescence properties with different ratios of Eu(THB)(THA)_2_Phen and NIR-780. When the donor/acceptor pair were co-assembled as one nanoprobe, intermolecular FRET occurred, and the phosphorescence intensity of Eu(THB)(THA)_2_Phen decreased (Fig. S18). Based on its good spectral properties, we chose Eu(THB)(THA)_2_Phen and IR-780 at the ratio of 50/7 as the nanoprobe for detecting HClO. Here, the RE complex Eu(THB)(THA)_2_Phen displayed efficient two-photon-sensitized and high-purity red emission. Moreover, it can emit characteristic fluorescence of Eu^3+^ under 780 nm excitation, and IR-780 can also be effectively excited by 780 nm wavelength. Therefore, we chose 780 nm excitation to observe the ratio changes in fluorescence. Due to the specific and fast recognition of hypochlorous acid by NIR-780, the absorption of IR-780 slowly decreased with the addition of HClO (Fig. 4b), and the luminescence intensity of Eu(THB)(THA)_2_Phen increased (Fig. 4c). The lifetime of Eu/NIR-NPs was 360.45 μs (decay time at 613 nm emission). After adding HClO, the lifetime increased to 698.32 μs (Fig. S19).

As shown in Fig. 4d and e, the addition of other ROS ($ \cdot $OH, ^1^O_2_, NO, ONOO^−^, t-BuOOH, H_2_O_2_) or substances (GSH, Cys, Glu, PO_4_^3−^, CO_3_^2−^, HS^−^, Cl^−^, SO_4_^2−^, NO_2_^−^, NO_3_^−^) did not cause a significant change in the fluorescence intensity of Eu/NIR-NPs in the aqueous solution. After adding HClO, the fluorescence of Eu/NIR-NPs changed obviously. The emission titration spectra of Eu/NIR-NPs towards HClO were measured (Fig. 4f). Under two-photon excitation at 780 nm, the change in the fluorescence intensity ratio *I*_613_/*I*_825_ with HClO concentration had a good linear relationship. The linear relationship between HClO and *I*_613_/*I*_825_ could be fitted as a function of equation (7),

(7) $$\begin
{equation}{I_{613}}/{I_{825}} = 0.44559{\rm{x}} + 0.01424,\end{equation}$$

with a correlation coefficient (R^2^) of 0.989 (Fig. 4g). The limit of detection (LOD) of HClO was calculated to be 95.60 nM with the formula 3s/k. Overall, these results clearly demonstrated that Eu/NIR-NPs acted as nanoprobes with excellent selectivity and anti-interference ability, and could effectively identify HClO using fluorescence signal ratio *I*_613_/*I*_825_ under two-photon excitation.

### Cytotoxicity and confocal fluorescence imaging in living cells

**Figure 5.**
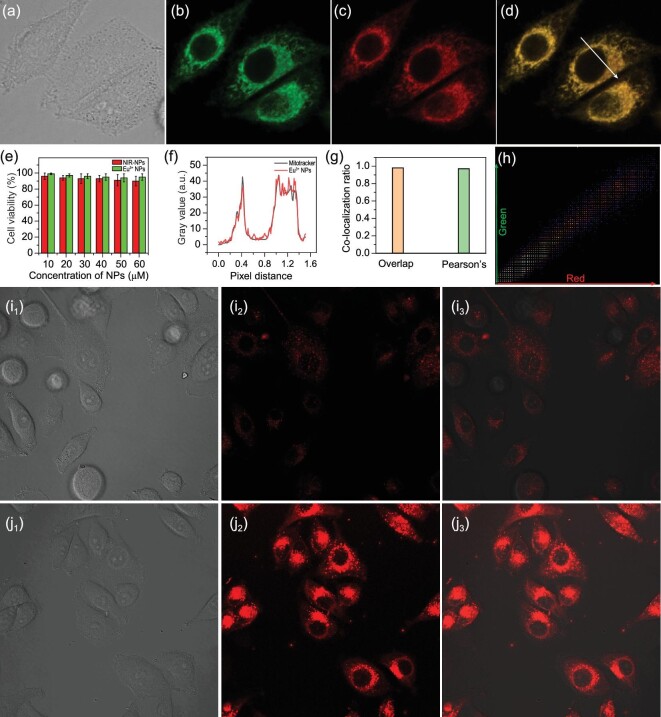
Confocal imaging photos of HeLa cells co-incubated with Eu^3+^-NPs (λ_ex_ = 405 nm; λ_em_ = 600–630 nm) and Mitotracker red (λ_ex_ = 641 nm; λ_em_ = 650–670 nm) for 30 min at 37°C. (a) Bright-field images; (b) Mitotracker red, λ_ex_ = 641 nm and λ_em_ = 650–670 nm; (c) Eu^3+^-NPs, λ_ex_ = 405 nm and λ_em_ = 600–630 nm. (d) Merged images. (e) MTT assays for HeLa cells incubated with Eu-NPs and NIR NPs. (f) Intensity profile of chosen regions (white arrow in Fig. 5d) across HeLa cells. (g) Pearson's coefficient and overlap coefficient. (h) Co-localization areas displayed of the red and green channels selected in Fig. 5d. Fluorescence images of HeLa cells incubated with (i) 10 μM of Eu/NIR-NPs for 1 h, (j) 10 μM of Eu/NIR-NPs for 1 h and further incubated for another 30 min with HClO. (1) Bright-field images. (2) Fluorescence channel images. (3) Merged images. λ_ex_ = 780 nm; λ_em _= 600–630 nm.

Since the nanoprobes Eu^3+^-NPs-0.5 are dispersed nanospheres with a particle size of less than 100 nm, and also show a positive potential of +35 eV, we expect that they can target mitochondria in living cells [46–48]. To confirm the hypothesis, we utilized co-localization experiments of Eu^3+^-NPs and Mitotracker red to describe the difference between their locations in mitochondria [49,50]. As shown in Fig. 5a–c, the living HeLa cells were co-stained with 5 μM Mitotracker red (a mitochondria dye, 30 min) and 10 μM Eu^3+^-NPs (30 min). The intensity profiles of Eu^3+^-NPs and Mitotracker red (640/665 nm) emissions in the linear region (white arrow in Fig. 5d) across HeLa cells varied in close synchrony (Fig. 5f). Besides, the fluorescence of Eu^3+^-NPs overlapped well with the red fluorescence of Mitotracker red (overlapping >98% and Pearson's correlation coefficient >90%) (Fig. 5g and h). All the above results verified that Eu^3+^-NPs possessed excellent ability to locate into mitochondria. Besides, the cytotoxicity was determined using MTT as an indicator. Even when Eu^3+^-NPs concentration was as high as 60 μM, 95% of the cells remained viable, which indicated that Eu^3+^-NPs had good biocompatibility and low cytotoxicity (Fig. 5e).

To further assess the biological application of Eu/NIR-NPs, two-photon fluorescence imaging for HClO detection was carried out in living HeLa cells. Under two-photon excitation, distinguishable fluorescence was generated in living cells and detected in real time. As described in Fig. 5i, cells treated only with Eu/NIR-NPs for 1 h at 37°C emitted a correspondingly weak luminescence emission in red channels under excitation at 780 nm. Nevertheless, when the same cells were treated with 50 μΜ of HClO for 0.5 h, a strong red luminescence signal was observed inside living cells (Fig. 5j). These results indicated that Eu/NIR-NPs could penetrate into cells and react with HClO in living cells.

## CONCLUSION

In summary, a new strategy was proposed to obtain Eu-NPs with SAIL characteristics. By systematically studying its self-assembly and optical properties in aqueous solution, it was found that the Eu^3+^-complex can be self-assembled into spherical nanoparticles in an aqueous solution while having the property of SAIL. Based on its efficient self-assembly in aqueous solution, we also studied its temperature-sensing properties by measuring the steady-state fluorescence and transient fluorescence changes at different temperatures. Experimental results indicated that Eu-NPs-0.5 as a fluorescence lifetime thermal sensor or luminescent sensor for temperature performed better than other reported thermal probes based on RE materials in terms of its maximum relative sensitivity. Finally, we successfully constructed a FRET system through co-assembly of the Eu^3+^-complex and IR-780. It was used as a ratiometric two-photon fluorescent probe to achieve sensitive and selective detection of hypochlorous acid in aqueous solution and living cells. We believe that the SAIL activity of the RE system proposed here offers a further step forward in the development of RE light conversion systems and their integrated applications in bioimaging and therapy.

## Supplementary Material

nwab016_Supplemental_File
