# Supplementary material for: Self-assembly-induced luminescence of Eu3+-complexes and application in bioimaging
Source: Natl Sci Rev. 2021 Jan 30;9(1):nwab016. doi: 10.1093/nsr/nwab016 (PMC8776545; doi:10.1093/nsr/nwab016)
Supplement: nwab016_Supplemental_File [file nwab016_supplemental_file.docx]

***Supporting Information***

**Self-assembly induced luminescence** **of Eu^3+^-complexes and application in bioimaging**

Ping-Ru Su,^1^ Tao Wang,^1^ Pan-Pan Zhou,^1^ Xiao-Xi Yang,^1^ Xiao-Xia Feng,^1^ Mei-Na Zhang,^1^ Li-Juan Liang,^1^ Yu Tang*^1,2^, and Chun-Hua Yan*^1^

^a^ State Key Laboratory of Applied Organic Chemistry, Key Laboratory of Nonferrous Metal Chemistry and Resources Utilization of Gansu Province, College of Chemistry and Chemical Engineering, Lanzhou University, Lanzhou 730000, P. R. China. Email: tangyu@lzu.edu.cn (Yu Tang). Phone: 86 931 8912552. Fax: 86 931 8912582. Email: [yan@lzu.edu.cn](mailto:caoj@lzu.edu.cn) (Chun-Hua Yan)

^b^ State Key Laboratory of Baiyunobo Rare Earth Resource Researches and Comprehensive Utilization, Baotou Research Institute of Rare Earths, Baotou 014030, P. R. China

**Contents**

1.Synthesis and experimental methods…………………………………..……….…S-3

2. Supporting Figures……………………………………………………..…...…...S-14

3. Supporting Tables………………..………………………………………..….....S-35

4. References………………………………………………………………...……..S-37

**1. Synthesis and experimental methods**

**Materials and Reagents:** All fluorescence data was collected by HORIBA fl-3. spectrophotometers. Mass spectra (ESI-MS) were obtained on a HP 5988A GC-MS spectrometer. ^1^H and ^13^C NMR spectra were collected on Varian INOVA 400 MHz spectrometers. Transmission electron microscopy (TEM) images and scanning electron microscopy (SEM) images were obtained from a Tecnai G2Tf20 instrument and Hitachi, S4700 FESEM system, respectively. The zeta potential and dynamic light scattering (DLS) of were measured on a Zetasizer (Nano ZS, Malvern, Worcestershire, U.K.). Matrix-Assisted Laser Desorption/ Ionization Time of Flight (MALDI-TOF)/MS were collected on Shimadzu MALDI-7090. Elemental analysis was conducted using an Elementar vario EL. All reagents including acetonitrile, dichloromethane (DCM), acetone, ethanol, triethylamine, carbazole, anhydrous AlCl_3_, lanthanide salts of Eu(NO_3_)_3_·6H_2_O, buffer solutions were purchased from J&K, Aldrich, Alfa, TCI and used as received. All solvents were analytical reagents.

**The self-assembly of Eu^3+^ complexes in water solution:** Eu^3+^ complexes were synthesized by the method in the previous literature,([1](#_ENREF_1)) and the detailed experimental steps were described in the *Supporting Information.* We adopted the strategy of using good solvents to dissolve the complexes, and then dispersing them in the poor solvent aqueous solution for self-assembly. A certain amount of Eu^3+^ complexes in organic solvent (such as acetone, THF, DMSO, DMF, Ethanol and MeCN) was added to aqueous solution with sonicating and continue sonicating the solution for 0.5 h. Subsequently, the prepared Eu-NPs were separated by centrifugation (12000 rpm, 5 min).

**Preparation of HClO probe (Eu/NIR-NPs):** Adding different ratios of Eu^3+^ complexes and IR-780 in acetone (100 µL, 0.4 mM) to 1 mL of aqueous solution with sonicating and continue sonicating the solution for 0.5 h. Subsequently, the prepared Eu/NIR-NPs were purified several times by dialysis using a semi-permeable membrane (molecular weight cut-off molecular weight 10000) in distilled water.

**Temperature measurement:** In order to study the feedback of the Eu-NPs to temperature changes, we selected Eu-NPs-0.5 (0.5 μM) as a thermometer and measured its steady-state fluorescence and transient-state fluorescence at different temperatures.

**Detection of HClO in water solution:** Reactive oxygen species (ROS) were prepared according to previous method.([2](#_ENREF_2)) The Eu/NIR-NPs was dissolved in double distilled water forming a storage solution with a concentration of 0.4 µM and utilized for all fluorescence measurement experiments. A stock solution of HClO (100 mM) and various ROS was also prepared with double distilled water. The UV-vis absorption spectrum and the luminescence spectrum were carried out in a HEPES buffer solution (10 mM, pH 7.4) at room temperature.

**In vitro imaging measurements:** HeLa cells were selected as cells for subsequent cell experiments. The cells were incubated in DMEM medium containing 15% fetal bovine serum and 5% CO_2_ at 37 °C. After culturing the cells for 24 hours, the cell co-localization experiment is processed by incubating cells with different concentrations of subcellular organelle positioning probes and Eu-NPs at 37°C for different times. The fluorescence signal is recorded under a Nikon confocal microscope. The intracellular detection of hypochlorous acid experiment is carried out by adding 50 μM of NaClO to the cells pre-incubated with the probe Eu/NIR-NPs. The luminescence signal is observed using a two-photon or single-photon microscope. Cell viability is tested in the presence of a range of Eu-NPs and Eu/NIR-NPs in the medium. First, Hela cells are seeded in 96-well plates to ensure that the cell concentration in each well was 10^5, and culture under 5% CO_2_ and 37 °C for 12 hours. Then add Eu-NPs or Eu/NIR-NPs of different concentrations to it, and incubate for 24h under the same conditions. After that, aspirate the medium, add 100μL MTT solution and incubate for 3h, and finally add 100μL DMSO solution. After shaking for ten minutes, the absorbance at 580 nm is measured on a microplate reader (Bio-TekELx800). Cytotoxicity was recorded as the percentage of cell survival and control cells.

**Synthesize of compound 1:** Carbazole (0.1 M, 16.72 g), 2-​chloro-​N,​N-​dimethylethanamine hydrochloride (0.15 M, 21.45 g) and sodium methoxide (0.2 M, 1.08 g) were dissolved in 50 mL methanol. After addition was complete, the mixture was stirred at 60 ℃ for 24 h. After cooling to room temperature, the mixture was extracted with methylene chloride. Organic layers were washed with brine, dried over MgSO_4_ and evaporated to afford a brown oil. The purified poduct was obtained as a brown solid after silica gel column chromatography (EtOAc /Methanol 20:1-10:1 as an eluent). Yield 70 %. *R_f_* = 0.27 (EtOAc /Methanol = 7/1 v/v). ^1^H NMR (400 MHz, CDCl_3_) δ 8.13 – 8.06 (m, 2H), 7.50 – 7.41 (m, 4H), 7.26 (d, J = 1.3 Hz, 1H), 7.24 – 7.20 (m, 1H), 4.53 – 4.23 (m, 2H), 2.74 (dd, J = 8.4, 7.1 Hz, 2H), 2.38 (s, 6H). ^13^C NMR (101 MHz,) δ 140.38 (s), 125.83 (s), 123.06 (s), 120.51 (s), 119.08 (s), 108.57 (s), 57.32 (s), 45.93 (s), 41.56 (s).

**Synthesize of compound 2:** Compound 1 (10 mmol, 2.38 g) was dissolved in 20 mL dry DCM, and then anhydrous AlCl_3_ (15 mmol, 2.0 g) was added. After the mixture solution was cold to 0 ℃ with ice salt bath, acetyl chloride (10 mmol, 0.785 g) in 20 mL dry DCM was added slowly into the solution. The mixture solution was stirred at room temperature until the reaction was completed by TLC (DCM:Methanol v/v=10:1). After reaction was finished, the mixture was quenched by 1.0 M HCl aqueous solution. Then, the product was extracted with EtOAc. Organic layers were washed with brine, dried over MgSO_4_ and evaporated to afford a brown oil. The purified poduct was obtained as a brown solid after silica gel column chromatography (DCM as an eluent). Yield 48 %. R_f_ = 0.46 (DCM/Methanol = 10/1 v/v). ^1^H NMR (400 MHz, CDCl_3_) δ 8.49 (s, 1H), 7.98 (t, J = 7.4 Hz, 2H), 7.62 (t, J = 9.5 Hz, 2H), 7.47 (d, J = 7.4 Hz, 1H), 7.25 (t, J = 7.4 Hz, 1H), 4.90 (s, 2H), 3.45 (dd, J = 8.0, 4.0 Hz, 2H), 2.91 (s, 6H), 2.57 (s, 3H). ^13^C NMR (101 MHz,) δ 197.76 (s), 143.15 (s), 141.06 (s), 129.02 (s), 126.63 (s),126.67 (s), 123.33 (s), 122.81 (s), 121.96 (s), 120.77 (s), 120.27 (s), 109.15 (s), 108.25 (s), 57.21 (s), 45.84 (s), 41.72 (s), 26.77 (s).

**Synthesize of compound 3:** Compound 2 (3.2 mmol, 0.894 g) was dissolved in 10 mL tert-Butanol and 10 mL dry THF, and then the mixture solution was cold to 0 ℃. Potassium t-Butoxide (18.75 mmol, 2.1 g) was added slowly to the mixture solution, and then solution was stirred at 0 ℃ for another 15 min. To the mixture solution, ethyl trifluoroethanoate (18mmol, 2.556 g) was added slowly and stirred for 12 h at room temperature. Then, the product was extracted with DCM. Organic layers were washed with brine, dried over MgSO_4_ and evaporated to afford a white solid. The purified poduct was obtained as a white solid after silica gel column chromatography (DCM/methanol= 20:1 as an eluent). Yield 46 %. R_f_ = 0.31 (DCM/Methanol = 10/1 v/v). ^1^H NMR (400 MHz, d-DMSO) δ 8.78 (d, J = 1.2 Hz, 1H), 8.29 (d, J = 7.8 Hz, 1H), 8.08 – 7.91 (m, 1H), 7.74 (dd, J = 16.6, 8.5 Hz, 2H), 7.48 (t, J = 7.7 Hz, 1H), 7.26 (t, J = 7.3 Hz, 1H), 6.43 (s, 1H), 4.85 – 4.60 (m, 2H), 3.32 (d, J = 7.1 Hz, 2H), 2.80 (d, J = 9.1 Hz, 6H).

**Synthesize of compound N,N,N-trimethyl-2-(3-(4,4,4-trifluoro-3-oxobutanoyl)-9H-carbazol-9-yl)ethan-1-aminium (HTHB) (4):**  Compound 3 (10 mmol, 3.76 g) was dissolved in 30 mL acetone and 0.5 mL Iodomethane was added slowly to the mixture solution. The solution was refluxed 3 h, resulting a yellow solid. The purified product was obtained by filtering without further Yield 94 %. ^1^H NMR (400 MHz, d-DMSO) δ 9.20 (d, J = 1.5 Hz, 1H), 8.41 (d, J = 7.7 Hz, 1H), 8.32 (dd, J = 8.8, 1.7 Hz, 1H), 7.92 (d, J = 8.8 Hz, 1H), 7.82 (d, J = 8.3 Hz, 1H), 7.64 (t, J = 7.3 Hz, 1H), 7.41 (t, J = 7.4 Hz, 1H), 7.26 (s, 1H), 5.09 – 4.90 (m, 2H), 3.83 – 3.65 (m, 2H), 3.29 (s, 9H). Electrospray ionization mass spectrometry (ESI-MS), calculated MS: 391.41, found MALDI-TOF/MS: m/z 391.72.

**Scheme S1.** Synthetic procedure of the compound 4.

**Synthesis of 9-amyl-carbazole (5).** To a solution of 9-H-carbazole (66.8 g, 400 mmol) and NaOH (24.0 g, 600 mmol) in acetone (300 mL) was refluxed for 2 hours. After cool to room temperature, 42 mL 1-bromopentane (66.0 g, 400 mmol) were added and refluxed for 24 hours. The solvent was removed by rotary evaporation and 200 mL water was added with stirring. The residual solid was filtered and recrystallized from ethanol/water to give colorless crystal product. Yield 80%, ^1^H NMR (400 MHz, CDCl_3_) δ 8.14 (t, J = 6.5 Hz, 2H), 7.62 – 7.37 (m, 4H), 7.35 – 7.24 (m, 2H), 4.32 (dd, J = 9.9, 4.2 Hz, 2H), 1.90 (d, J = 3.1 Hz, 2H), 1.40 (d, J = 3.3 Hz, 4H), 1.01 – 0.78 (m, 3H). ^13^C NMR (101 MHz, CDCl_3_) δ 140.57 (s), 125.68 (s), 122.95 (s), 120.46 (s), 118.81 (s), 108.77 (s), 43.17 (s), 29.56 (s), 28.81 (s), 22.62 (s), 14.09 (s).

**Synthesis of 3-acetyl-9-amyl-carbazole (6):** To a solution of 9-amyl-carbazole (9.48 g, 40 mmol) in dichloromethane (100 mL) was rapidly added AlCl_3_ (10.4 g, 80 mmol) with stirring. After cooling to 0 ℃, a solution of acetic anhydride (4.08 g, 40 mmol) in 30 mL of dichloromethane was added dropwise over 30 min under vigor stirring. After stirring overnight at room temperature, a large amount of water and HCl was slowly added into the mixture and extracted with dichloromethane twice, washed by 1M NaHCO_3_ and water. The combined organic phase was dried over anhydrous MgSO_4_ and then filtered. The organic solvent was completely removed by rotary evaporation. The residue was purified by column chromatograph using petroleum ether and ethyl acetate as eluent to give the purified title compound. Yield 70%, ^1^H NMR (400 MHz, CDCl_3_) δ 8.72 (s, 1H), 8.17 – 8.05 (m, 2H), 7.49 (t, J = 7.6 Hz, 1H), 7.41 (dd, J = 8.3, 1.1 Hz, 1H), 7.36 (dd, J = 8.6, 4.9 Hz, 1H), 7.28 (t, J = 7.4 Hz, 1H), 4.27 (q, J = 7.0 Hz, 2H), 2.70 (s, 3H), 1.85 (dd, J = 6.8, 4.3 Hz, 2H), 1.34 (d, J = 3.5 Hz, 4H), 0.86 (t, J = 7.0 Hz, 3H). ^13C^ NMR (101 MHz,) δ 197.70 (s), 143.28 (s), 141.22 (s), 128.82 (s), 126.49 (s), 126.47 (s) 123.27 (s), 122.68 (s), 121.93 (s), 120.68 (s), 120.02 (s), 109.32 (s), 108.35 (s), 43.38 (s), 29.43 (s), 28.72 (s), 26.71 (s), 22.53 (s), 14.01 (s).

**Synthesis of 4,4,4-trifluoro-1-(9-amylcarbazole-3-yl)-1,3-butanedione (HTHA) (7):** To a solution of ethyl trifluoroacetate (1.71 g, 12 mmol) in anhydrous THF (20 mL) was added t-BuOK (3.33 g, 15 mmol). A solution of 3-acetyl-9-hexyl-carbazole (2.79 g, 10 mmol) in 10 mL of anhydrous EtOH was added dropwise over 5 min, and then stirring for 6 hours at room temperature. After 3 M HCl was added to the mixture with stirring, the mixture was extracted with dichloromethane and the organic layer was collected. The organic layer was wished with brine, dried over anhydrous MgSO_4_ and filtered. The organic solvent was completely removed by rotary evaporation. The residue was purified by chromatography on a silica gel with gradient petroleum ether and ethyl acetate to give the yellowish crystal of the title compound. Yield 76%, ^1^H NMR (400 MHz, CDCl_3_) δ 8.69 (d, J = 3.7 Hz, 1H), 8.13 (d, J = 7.8 Hz, 1H), 8.09 – 7.98 (m, 1H), 7.53 (dd, J = 11.3, 3.9 Hz, 1H), 7.48 – 7.36 (m, 2H), 7.32 (t, J = 7.4 Hz, 1H), 6.68 (s, 1H), 4.36 – 4.15 (m, 2H), 1.98 – 1.80 (m, 2H), 1.35 (td, J = 7.1, 3.6 Hz, 4H), 0.88 (t, J = 6.7 Hz, 3H). ^13^C NMR (101 MHz, ) δ 187.51 (s), 175.41 (s), 175.06 (s), 144.08 (s), 141.26 (s), 127.00 (s), 125.67 (s), 123.40 (d, J = 17.1 Hz), 122.93 (s), 121.40 (s), 120.85 (s), 120.57 (s), 119.14 (s), 116.33 (s), 109.55 (s), 109.00 (s), 91.76 (s), 43.50 (s), 29.42 (s), 28.71 (s), 22.51 (s), 13.99 (s). Electrospray ionization mass spectrometry (ESI-MS), calculated MS: 375.14, found [M+H]^+^ = 376.22 and [M+ H_2_O]^+^ = 394.23 (Fig. S2 ).

**Scheme S2.** Synthetic procedure of the compound 7.

**Synthesis of Eu^3+^-complex (Eu(THB)(THA)_2_Phen):** The complex was synthesized according to previous work [1]. Compound 4 (1 mM, 0.518 g), compound 7 (2 mM, 0.750 g) and 1,10 o-phenanthroline (1 mM, 0.180 g) were dissolved in 10 mL Ethanol. The solution was adjusted pH to 8.0 with 1M NaOH (3 mL), resulting a clear solution. And then, the solution of Eu(NO_3_)_3_.6H_2_O (1 mM, 0.446 g) was dropped into the above solution, forming a light yellow turbid solution, which was stirred at 60 ℃ for another 12 h. The product was vacuum dried after filtering and washing with water and Ethanol for several times. Yield 74%. MS: m/z: 1471.42. (Figure S28-S30) Anal. Calcd. for C_74_H_66_O_6_N_6_F_9_IEu: C, 56.07; H, 4.20; N, 5.30, Eu, 9.59 %; found C, 58.83, H, 4.33, N, 4.91, Eu, 9.83 %.

**Scheme S3.** Synthetic procedure of the compound Eu^3+^-complex Eu(THB)(THA)_2_Phen.

**Synthesis of compound 8:** Compound 8 was synthesized according to a slight modification of the literature procedure[[2](#_ENREF_2)] .

**Synthesis of IR-780:** Compound 8 (5 mmol, 4.92 g) was dissolved in 30 mL acetone and 0.5 mL Iodomethane was added slowly to the mixture solution. The solution was refluxed 3 h, resulting a green solid. The purified product was obtained by filtering without further Yield 90 %.

**Scheme S4.** Synthetic procedure of the compound 8.

**Synthesis of 4-(4,6-dichloro-1,3,5-triazin-2-yl)-N,N-diethylaniline (9)^[4]^:** Cyanuric chloride (9.88 g, 0.054 mmol) and *N, N*-diethylaniline (16 g, 0.108 mmol) were mixed in a round bottom flask. Then the reaction mixture was refluxed at 75 ⁰C for 8 h. The completion of the reaction was monitored by TLC. The reaction mixture was then cooled to room temperature and it was slowly poured into a beaker containing hexane, the brownish yellow solid was obtained. The formed solid was washed with 200 ml of hexane: dichloromethane (1:1). Washed the crude yellow solid with hexane several times to get pure product. Then the solid was filtered and dried under vacuum. Appearance: yellow solid. TLC: (Ethylacetate: Hexane, 10:90 v/v), R_f_ value: 0.5; Yield: 70%, ^1^H NMR (CDCl_3_, 400 MHz): δ (ppm) 8.29 (d, 2H, ArH), 6.65 (d, 2H, ArH), 3.45 (q, 4H, -CH_2_), 1.21(t, 6H, -CH_3_).

**Synthesis of 4-(4,6-bis(3,5-dimethyl-1H-pyrazol-1-yl)-1,3,5-triazin-2-yl)-N, N-diethylaniline (dpqt, 10):** The compound was synthesized according previous work [3]. 3,5-Dimethylpyrazole (970 mg, 0.095 mol) was dissolved in dry THF (60 ml) and potassium metal (271 mg, 6.96 mmol) was added at room temperature and the solution was refluxed at 70 ⁰C under N_2_ atmosphere. The complete colorless solution appeared after 2 h, then the reaction mixture was cooled to room temperature and 4-(4,6-dichloro-1,3,5-triazin-2-yl)-N,N-diethylaniline (ii) (1 g, 3.365 mmol) was added and refluxed for 6 h. The reaction mixture was kept at 4 ⁰C for overnight to get dpqt as pale yellow crystal like solid, the excess THF solvent was slowly decanted and the compound was washed with THF and hexane. The solid was dried under vacuum. Appearance: Yellow solid. TLC: (CH_3_OH:CH_2_Cl_2_, 10:90) R_f_ value: 0.3. Yield: 70%.^1^H NMR (400 MHz, CDCl_3_) δ 8.37 (d, J = 9.1 Hz, 2H), 6.73 (d, J = 9.2 Hz, 2H), 6.08 (s, 2H), 3.47 (q, J = 7.1 Hz, 4H), 2.86 (s, 6H), 2.35 (s, 6H), 1.24 (t, J = 7.1 Hz, 6H).


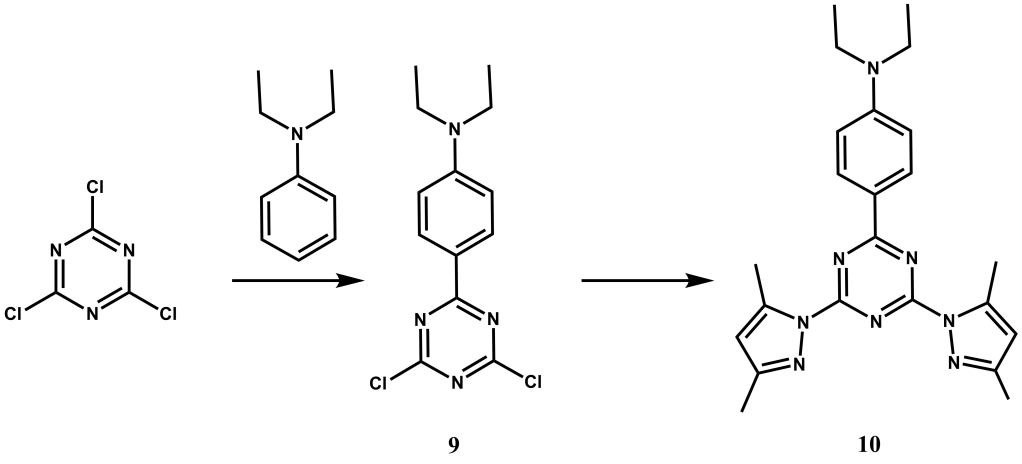


**Scheme S5.** Synthetic procedure of the ligand 10.

**Synthesis of Eu^3+^-complex (Eu(tta)_3_(dpqt)):** The complex Eu(tta)_3_(dpqt) was synthesized according to previous work [4]. Compound 10 (0.1mmol, 0.0416 g), 2-Thenoyltrifluoroacetone (0.3 mmol, 0.0667 g) were dissolved in 10 mL THF. The solution was adjusted pH to 8.0 with 1M NaOH (0.3 mL), resulting a clear solution. And then, the solution of Eu(NO_3_)_3_·6H_2_O (0.1mM, 0.0446 g) was dropped into the above solution, forming a light yellow turbid solution, which was stirred at 60 ℃ for another 12 h. After evaporation of the solvent, the residue was redissolved in a small amount of diethyl ether. Addition of n-hexane to the solution led to the precipitation of the ternary complex of [Eu(tta)_3_(bpqt)] as an orange powder.

**Scheme S6.** Synthetic procedure of the compound Eu^3+^-complex Eu(tta)_3_(dpqt).

**Synthesis of Eu^3+^-complex (Eu(THA)_3_(dpqt)):** Compound 10 (0.1mmol, 0.0416 g), compound 7 (0.3 mmol, 0.1126 g) were dissolved in 10 mL THF. The solution was adjusted pH to 8.0 with 1M NaOH (0.3 mL), resulting a clear solution. And then, the solution of Eu(NO_3_)_3_·6H_2_O (0.1 mM, 0.0446 g) was dropped into the above solution, forming a light yellow turbid solution, which was stirred at 60 ℃ for another 12 h. After evaporation of the solvent, the residue was redissolved in a small amount of diethyl ether. Addition of n-hexane to the solution led to the precipitation of the ternary complex of [Eu(tta)_3_(bpqt)] as an orange powder.

**Scheme S7.** Synthetic procedure of the compound Eu^3+^-complex Eu(THA)_3_(dpqt).

**Synthesis of Eu^3+^-complex (Eu(THA)_3_Phen):** The complex was synthesized according our previous work [5]. Compound 7 (0.31mmol, 0.1126 g) and 1,10 o-phenanthroline (0.1 mM, 0.0180 g) were dissolved in 10 mL Ethanol. The solution was adjusted pH to 8.0 with 1M NaOH (0.3 mL), resulting a clear solution. And then, the solution of Eu(NO_3_)_3_.6H_2_O (1mM, 0.446 g) was dropped into the above solution, forming a white turbid solution, which was stirred at 60 ℃ for another 12 h. The product was vacuum dried after filtering and washing with water and Ethanol for several times.

**Scheme S8.** Synthetic procedure of the compound Eu^3+^-complex Eu(THA)_3_Phen.

**2. Supporting Figures**


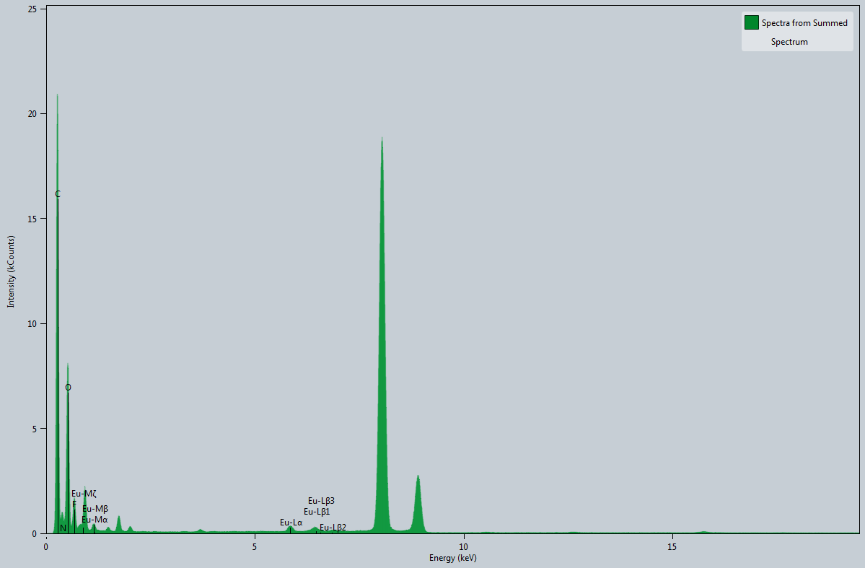


**Figure S1.** The EDS spectrum of Eu-NPs.


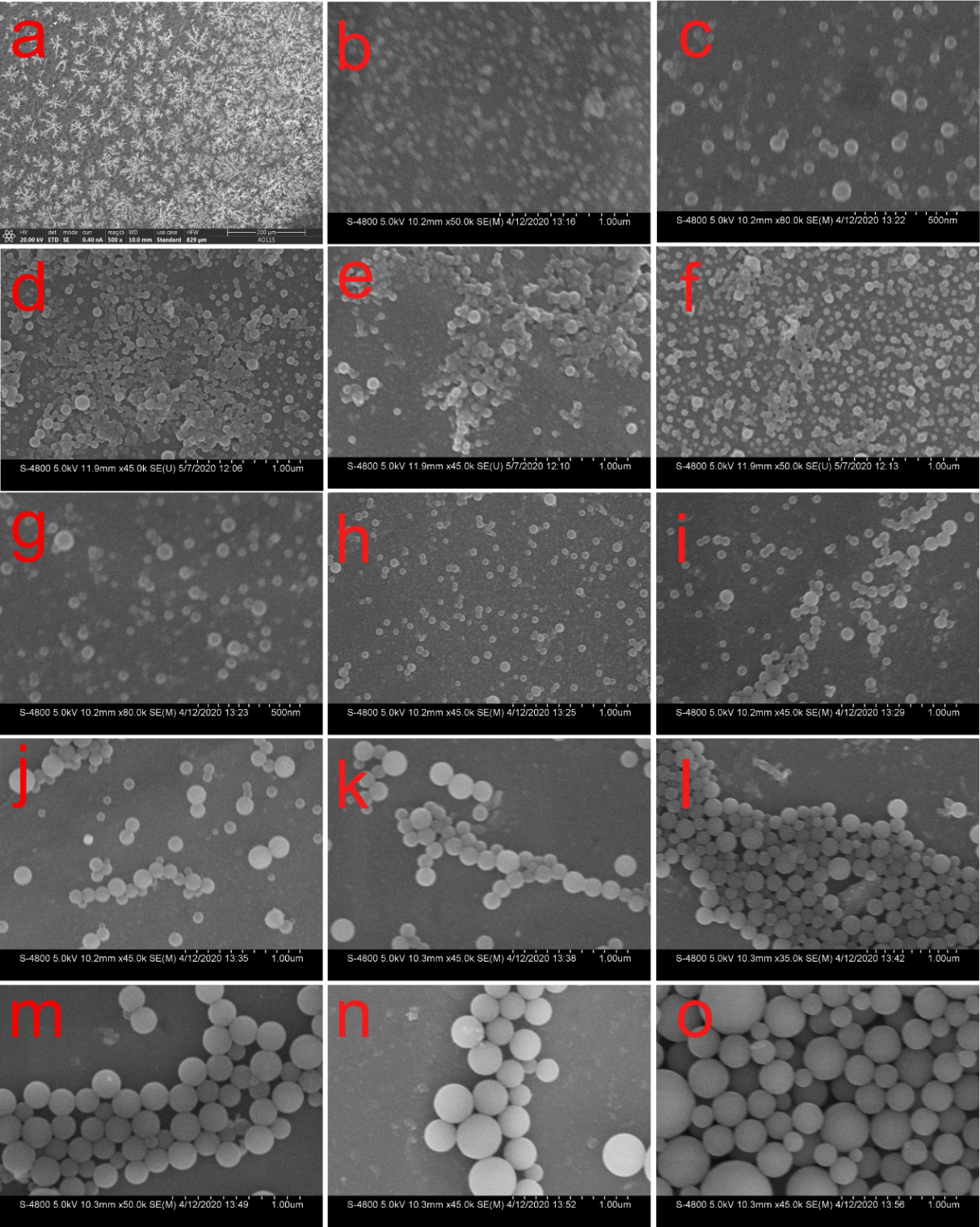


**Figure S2.** SEM images of Eu-NPs at different concentration of Eu^3+^ complexes (a) 0.01 mM, (b) 0.02 mM, (c) 0.04 mM, (d) 0.06 mM, (e) 0.08 mM, (f) 0.1 mM, (g) 0.2 mM, (h) 0.3 mM, (i) 0.4 mM, (j) 0.5 mM, (k) 0.6 mM, (l) 0.7 mM, (m) 0.8 mM, (n) 0.9 mM and (o) 1.0 mM.


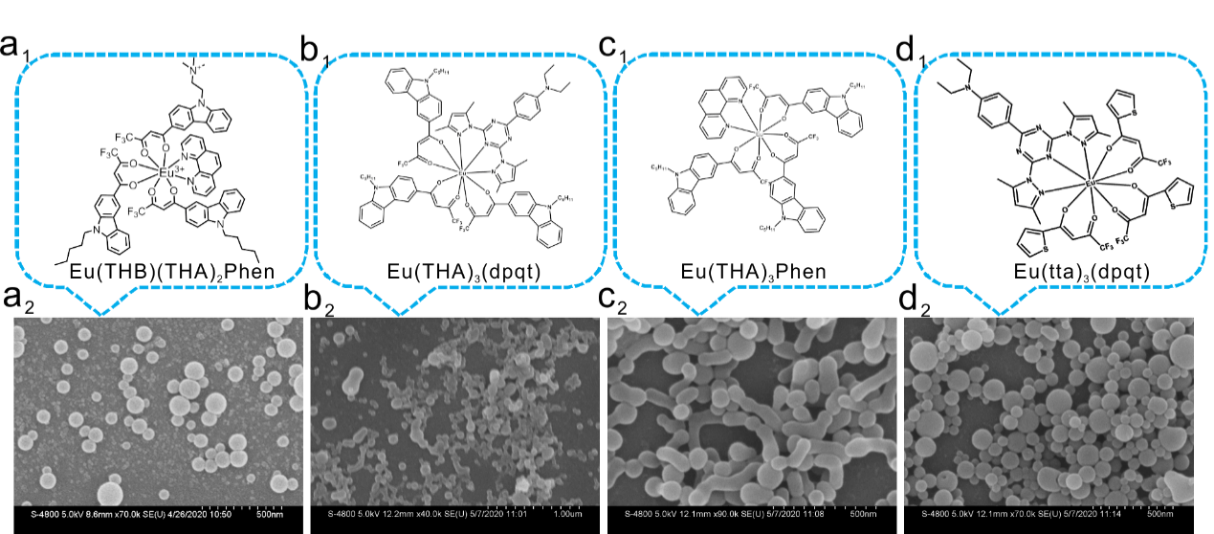


**Figure S3.** (a_1_-d_1_) The structure of different Eu^3+^-complexes. (a_2_-d_2_) SEM images of the self-assembly of the corresponding Eu^3+^-complexes in Figure (a_1_-d_1_).


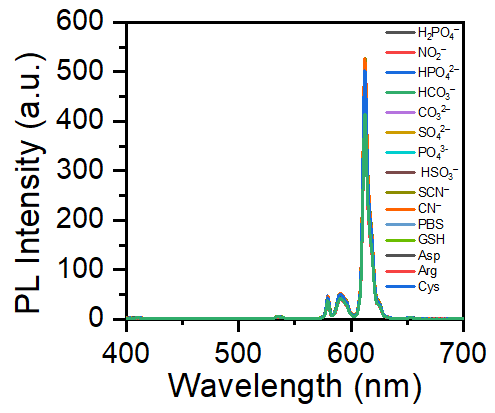


**Figure S4.** Fluorescence spectra of Eu-NPs-1.0 (10 μM) after adding of all kinds of 100 μM of H_2_PO_4_^-^, NO_2_^-^, HPO_4_^2-^, HCO_3_^-^, CO_3_^2-^, SO_4_^2-^, PO_4_^3-^, HSO_3_^-^, SCN^-^, CN^-^, PBS, GSH, Asp, Arg, Cys in water solution.


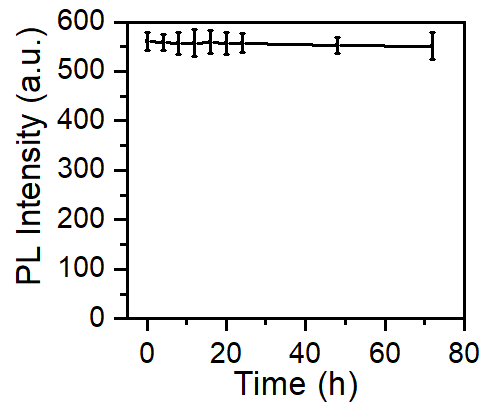


**Figure S5.** Fluorescence spectra of Eu-NPs-1.0 (10 μM) at different time.


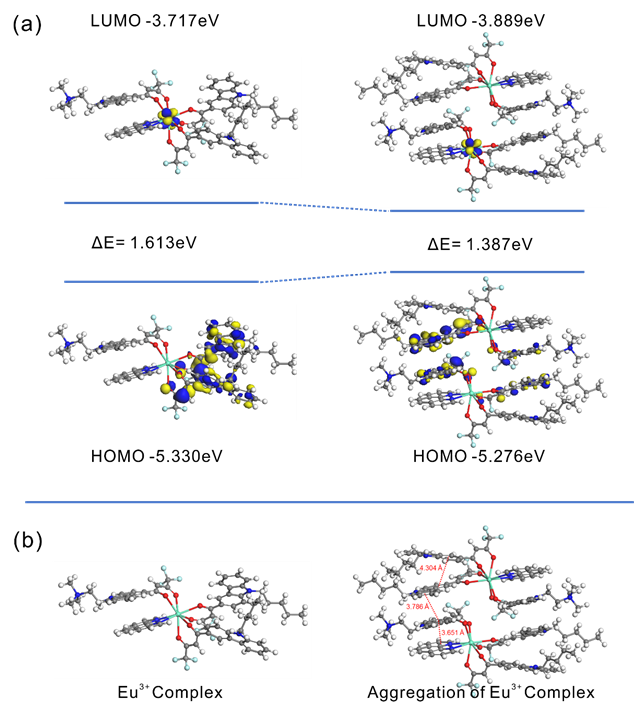


**Figure S6.** (a) The optimized geometries and stacking forms of the Eu(THB)(THA)_2_Phen. (b) The interfacial plots of the orbitals HOMO-LUMO energy gaps for Eu(THB)(THA)_2_Phen and aggregation of Eu(THB)(THA)_2_Phen.


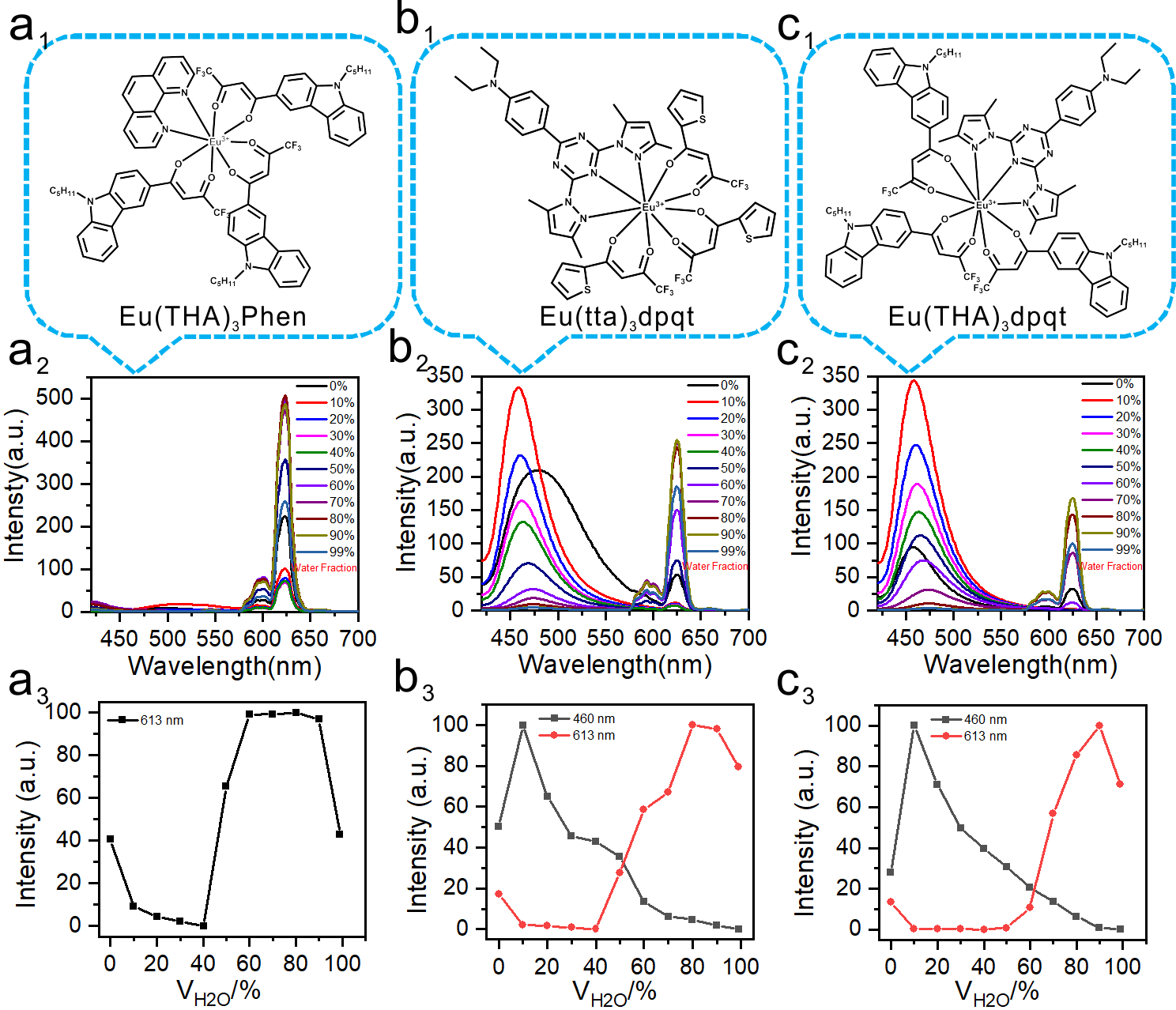


**Figure S7.** (a_1_-c_1_) The structure of different Eu^3+^-complexes. (a_2_-c_2_) Photoluminescence spectra of Eu^3+^-complexes (50 μM) in acetone/water mixtures. (a_3_-c_3_) The normalized luminescence intensity of Eu^3+^ complexes (50 μM) in acetone/water mixtures.


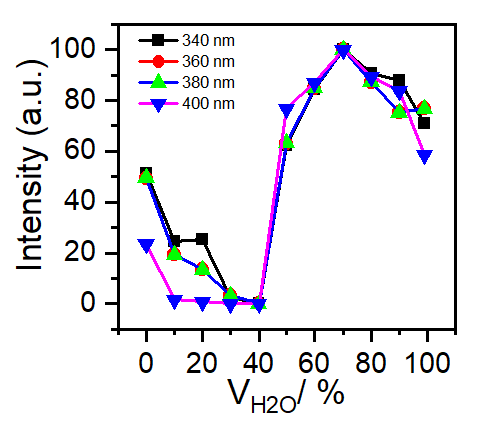


**Figure S8.** The normalized luminescence intensity of Eu(THB)(THA)_2_Phen (50 μM) in acetone/water mixtures with different excitation.


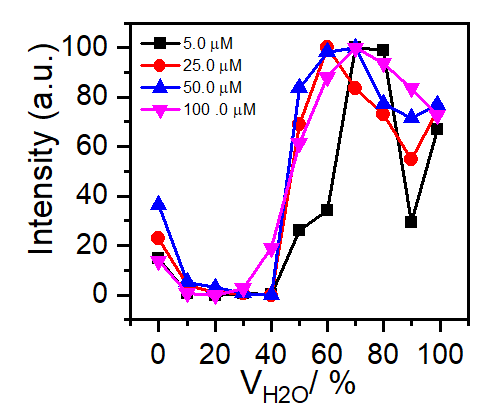


**Figure S9.** The normalized luminescence intensity of Eu(THB)(THA)_2_Phen in acetone/water mixtures with different concentration.


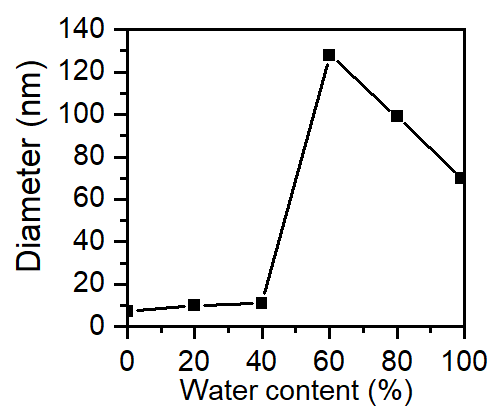


**Figure S10.** Hydrodynamic particle size distribution of Eu-NPs (100 μM) at different water content measured by DLS.


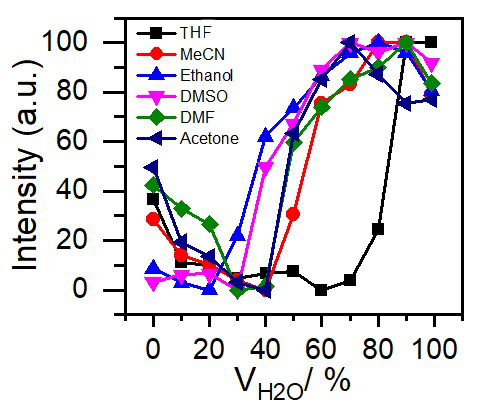


**Figure S11.** The normalized luminescence intensity of Eu(THB)(THA)_2_Phen (50 μM) in different organic solvent/water. Ex: 390 nm.


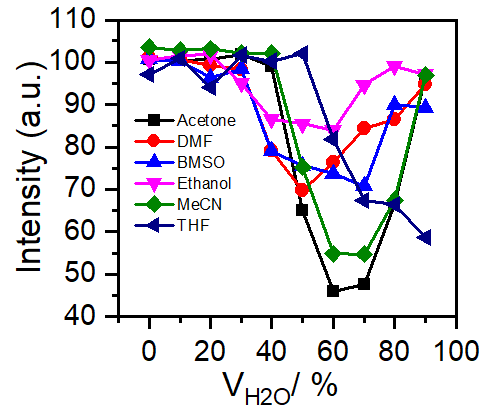


**Figure S12.** Optical transmittance of Eu(THB)(THA)_2_Phen (50 μM) in different organic solvent/water at 25 °C.


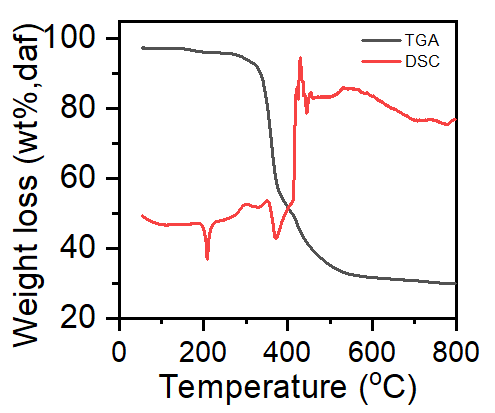


**Figure S13.** TG-DSC spectrum of Eu-NPs-0.5 in 10 K/min tests.


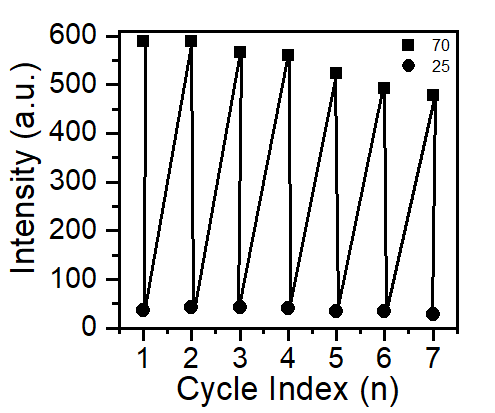


**Figure S14.** Fluorescence emission intensity changes of Eu-NPs-0.5 by alternately heating water solution. Excitation wavelength: 390 nm.


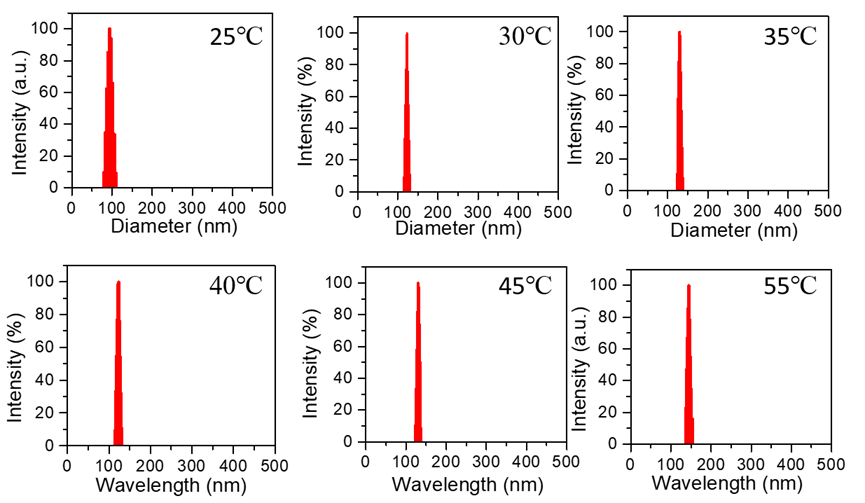


**Figure S15.** Particle distribution of Eu-NPs-0.5 at different temperature (25℃ to 55℃) in water solution measured by DLS.


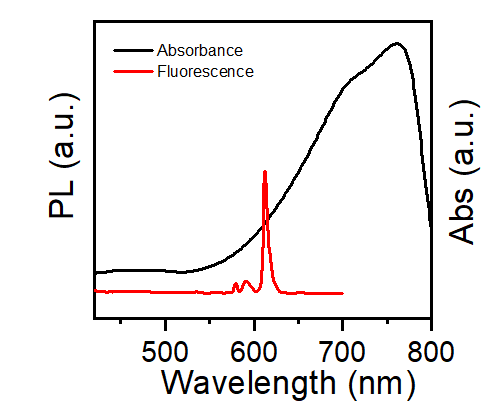


**Figure S16.** The UV-Vis spectrum of IR-780 and luminescence spectrum of Eu(THB)(THA)_2_Phen in water media.


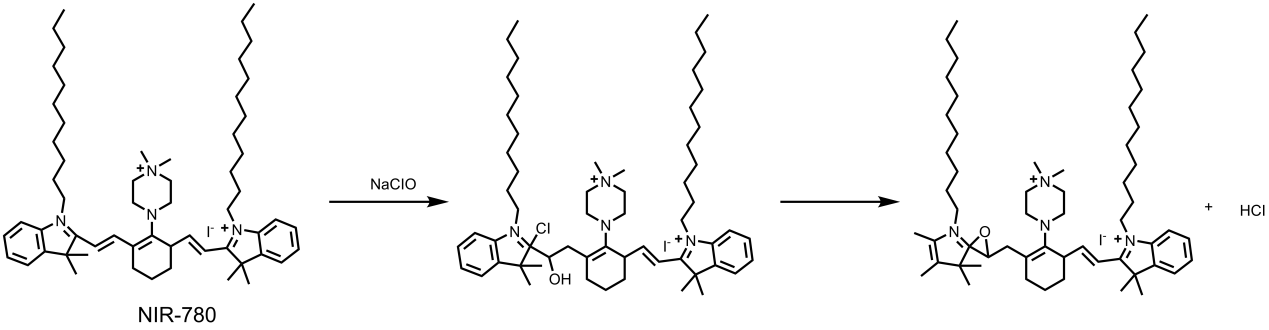


**Figure S17.** The proposed sensing mechanism of NIR-780.


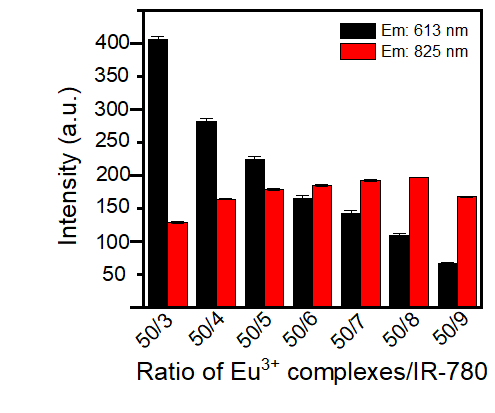


**Figure S18.** The fluorescence intensity of Eu/NIR-NPs at emission of 613 nm and 825 nm with different ratio of IR-780 and Eu(THB)(THA)_2_Phen in water media.


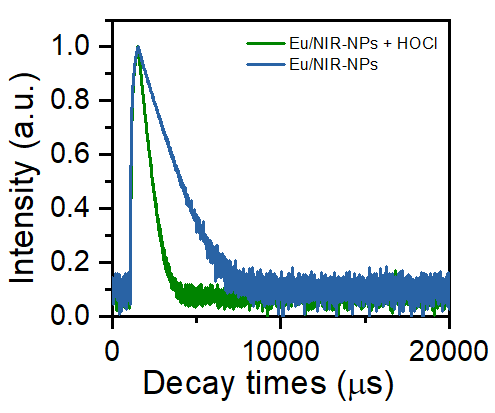


**Figure S19.** The decay curves at the wavelength of 613 nm for Eu/NIR-NPs and Eu/NIR-NPs + HOCl at different temperature.


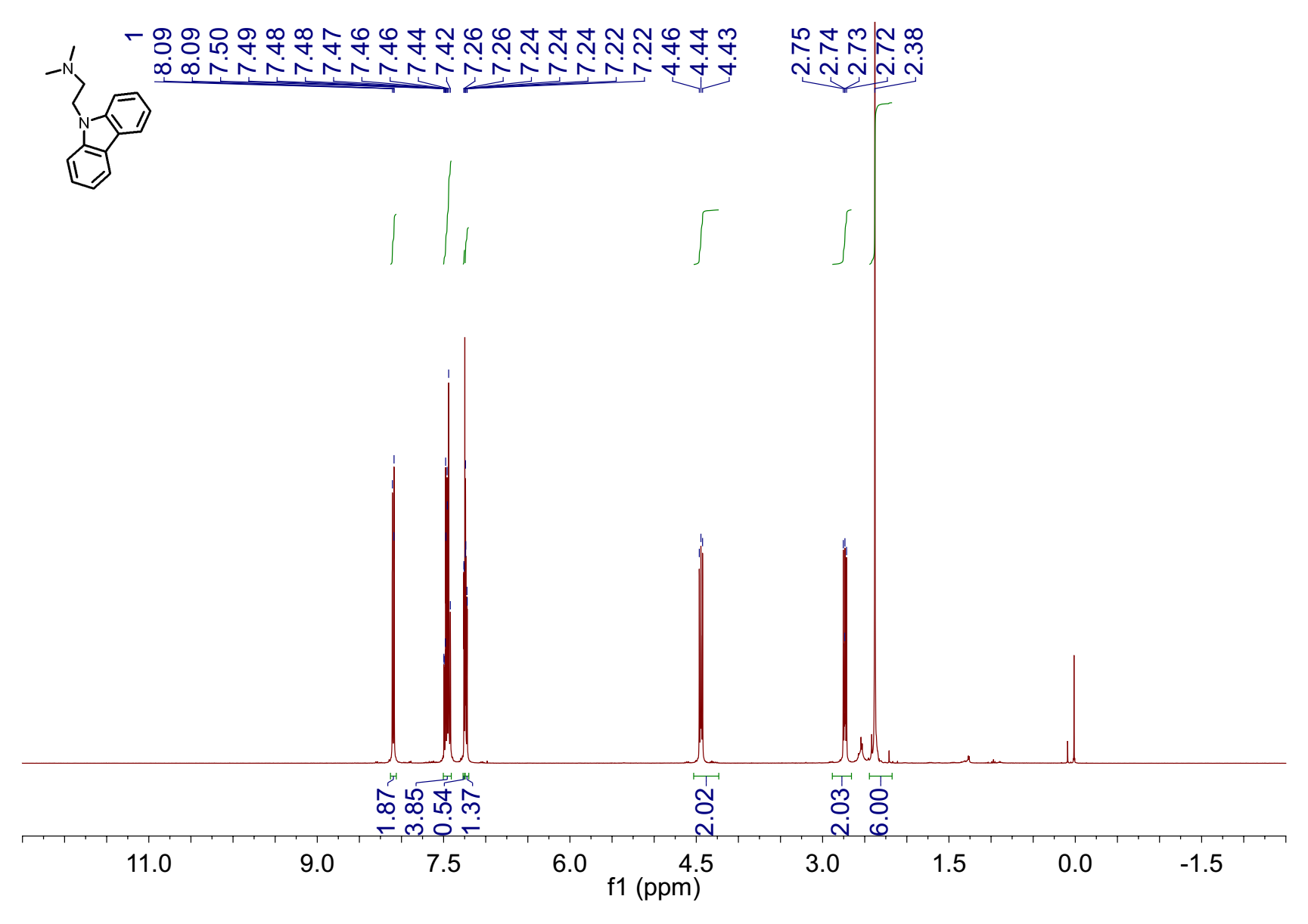


**Figure S20.** ^1^H NMR spectrum of compound 1 in CDCl_3_.


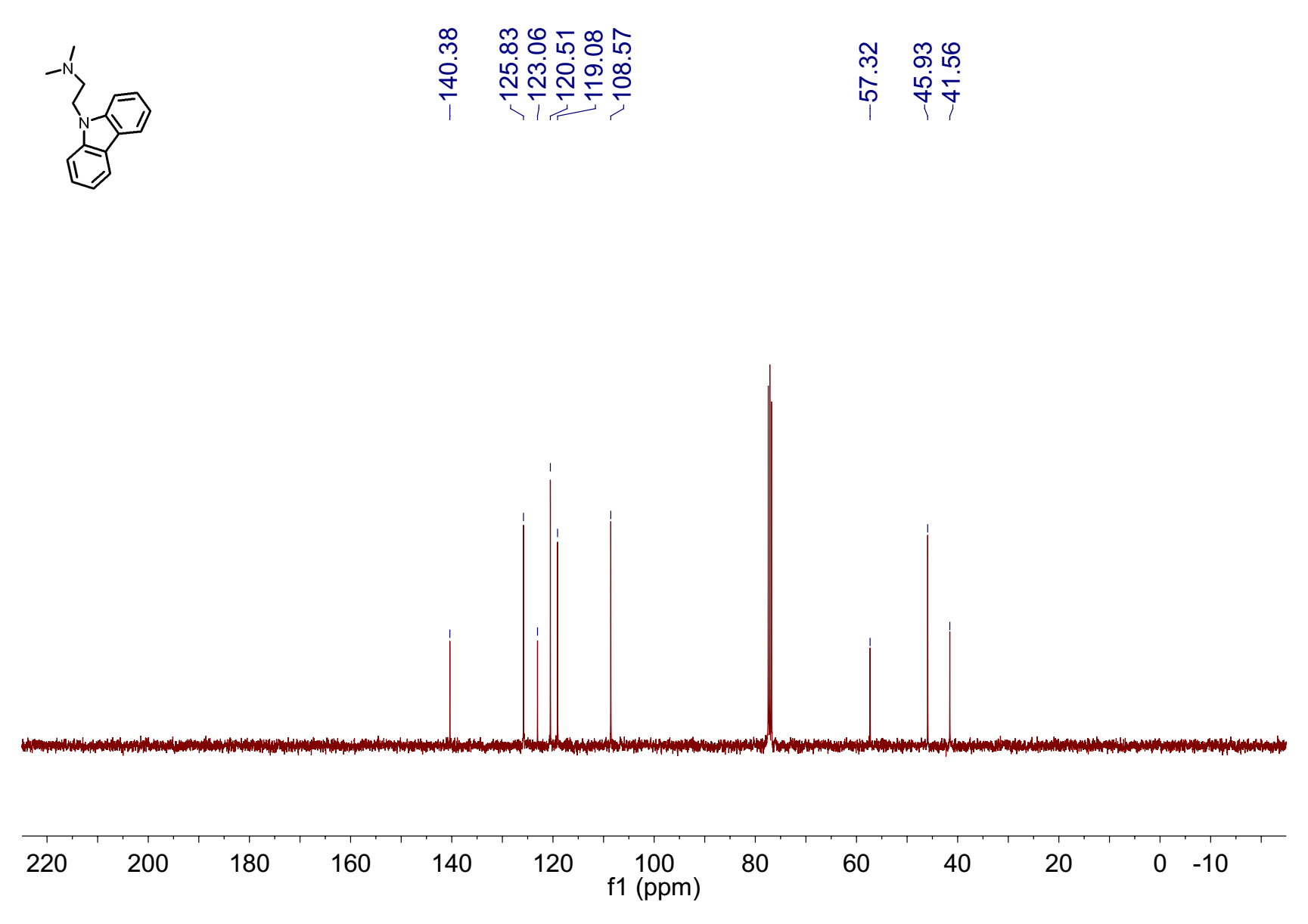


**Figure S21.** ^13^C NMR spectrum of compound 1 in CDCl_3_.


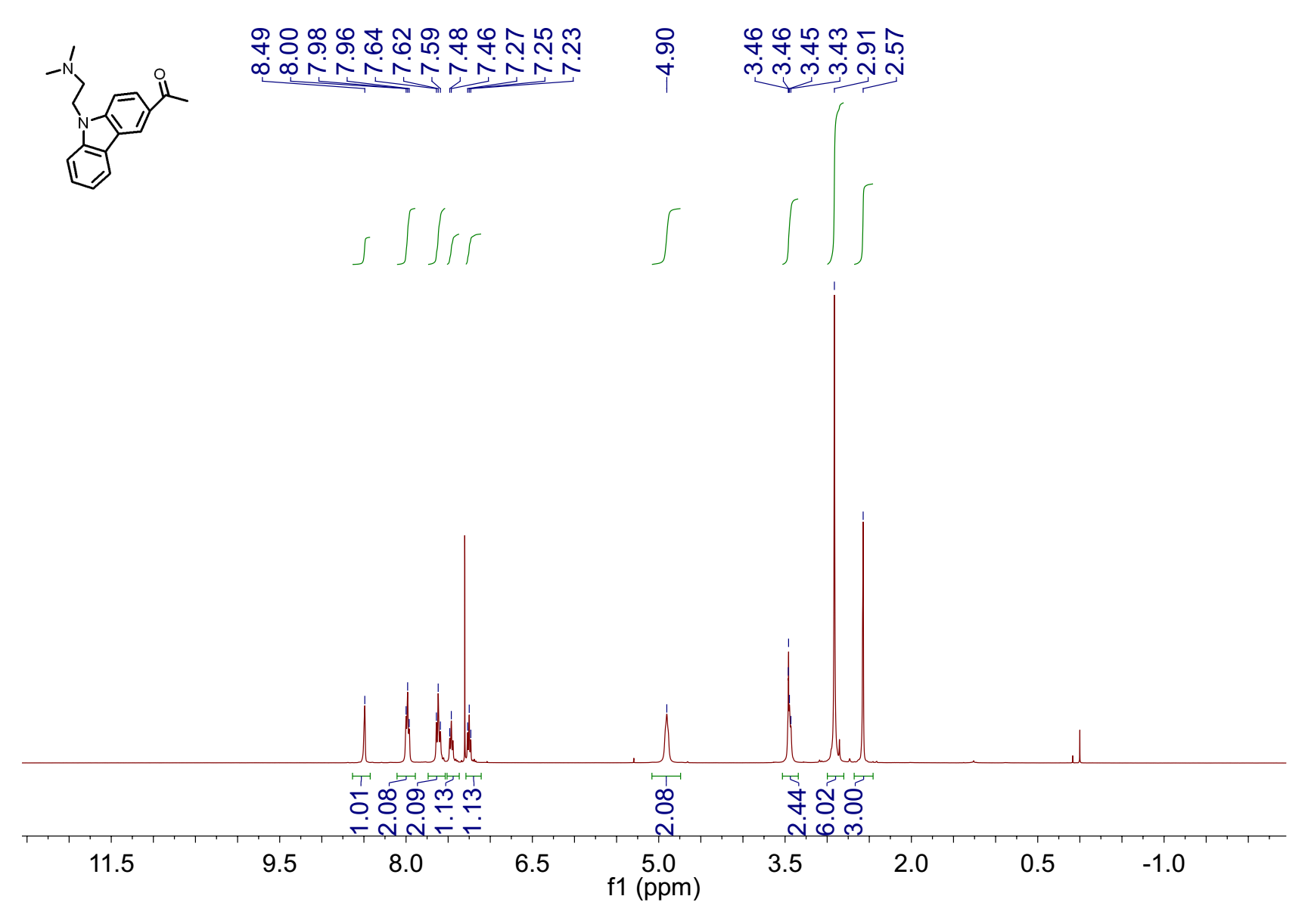


**Figure S22.** ^1^H NMR spectrum of compound 2 in CDCl_3_.


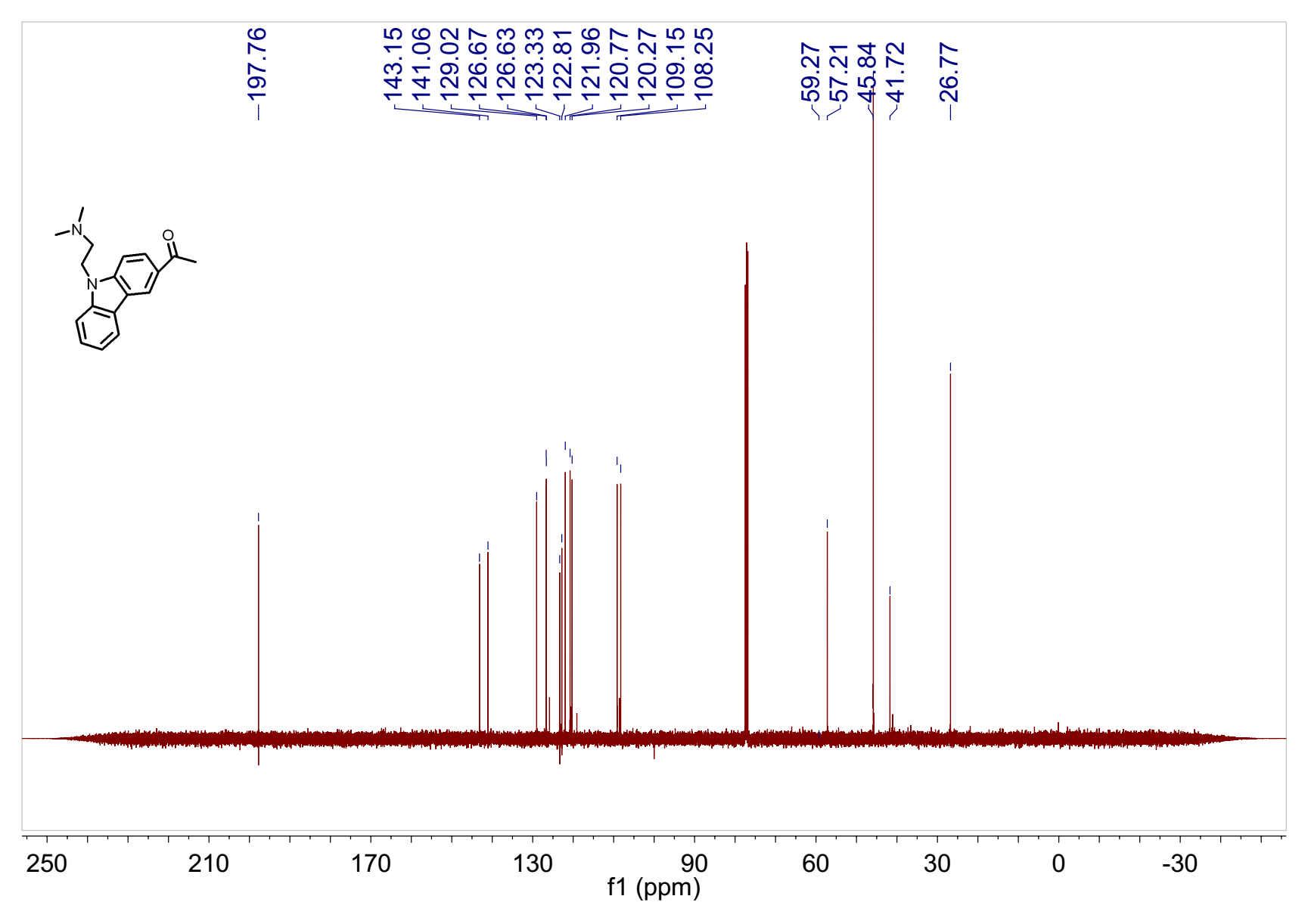


**Figure S23.** ^13^C NMR spectrum of compound 2 in DMSO.


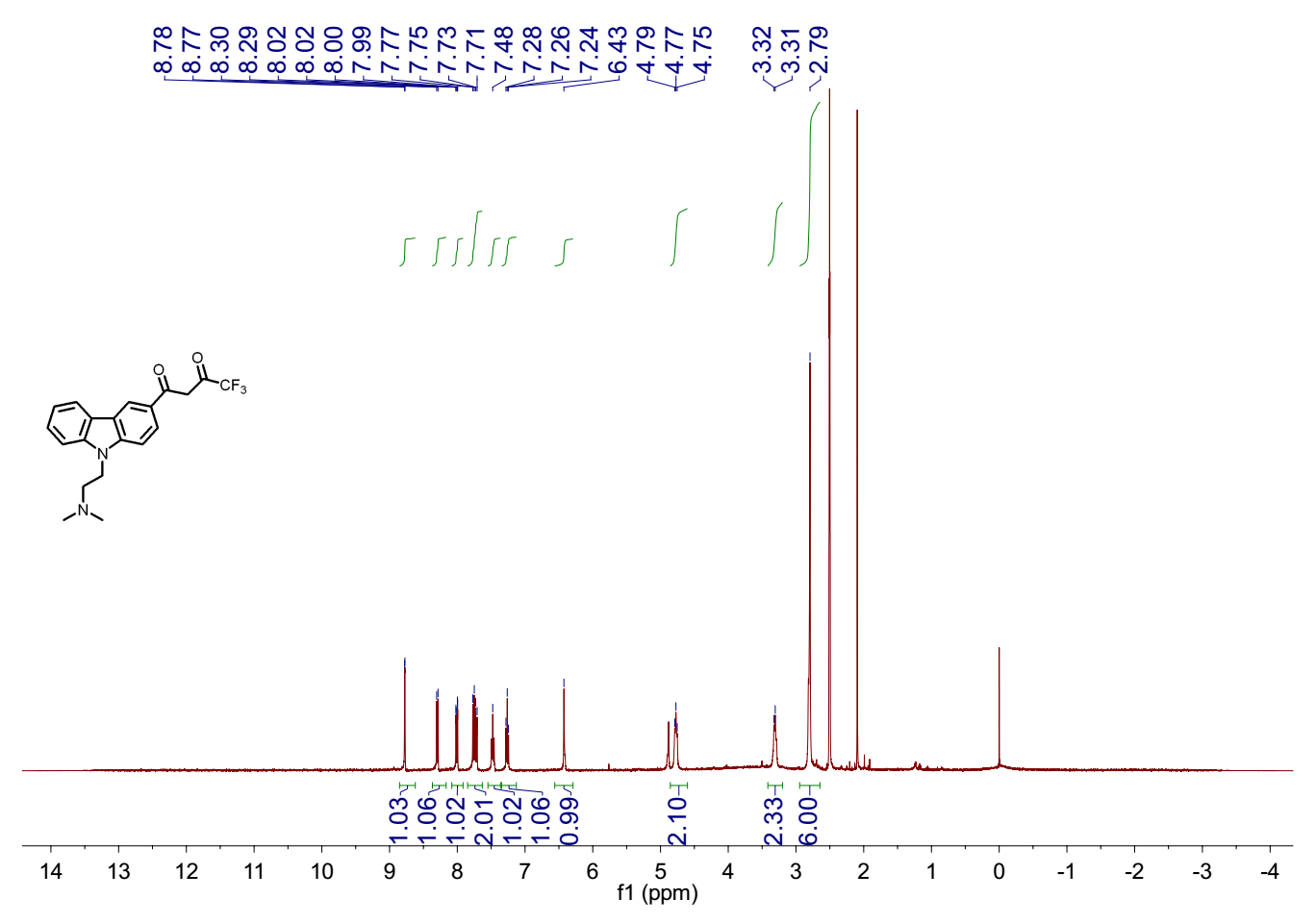


**Figure S24.** ^1^H NMR spectrum of compound 3 in DMSO.


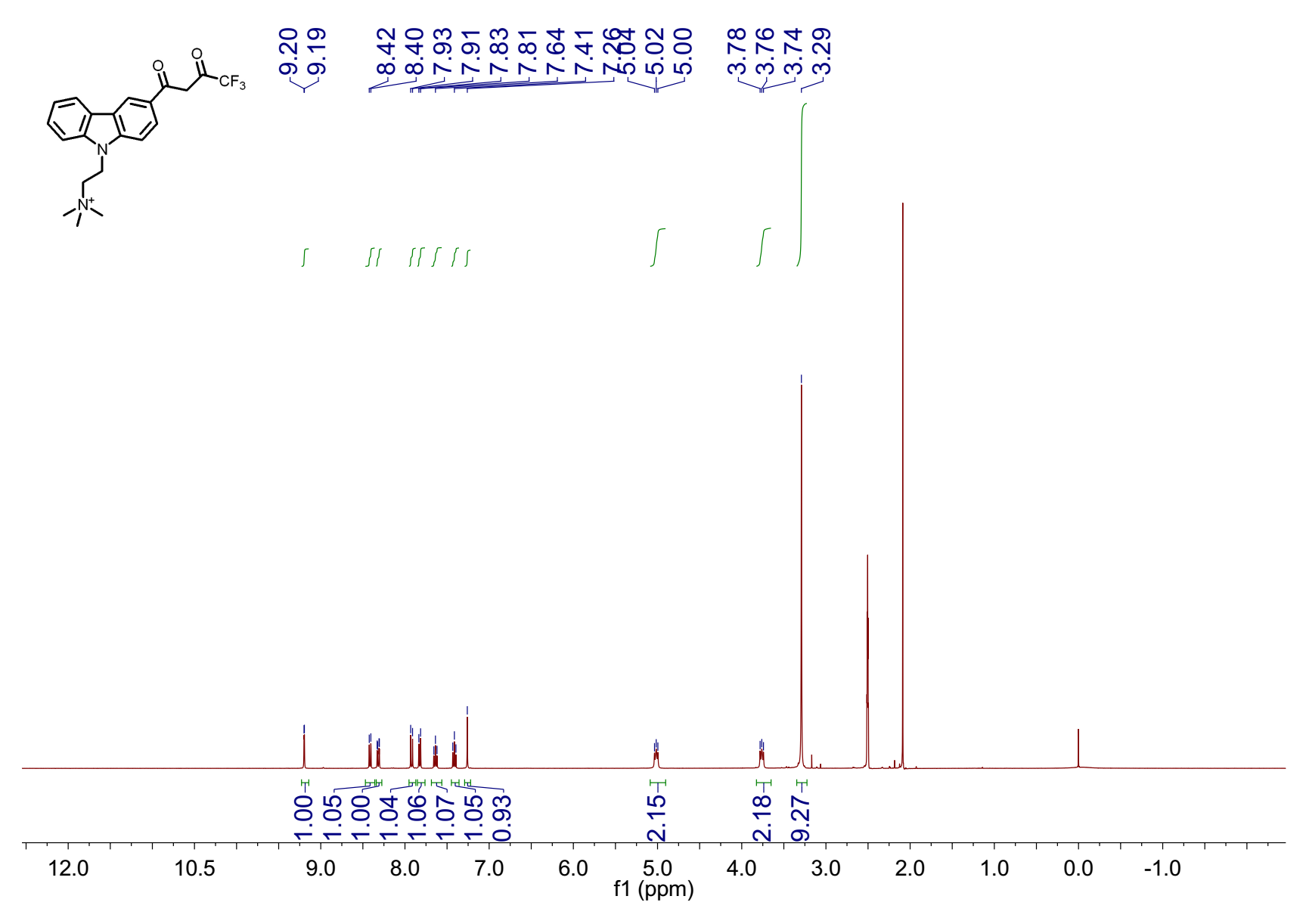


**Figure S25.** ^1^H NMR spectrum of compound 4 in DMSO.


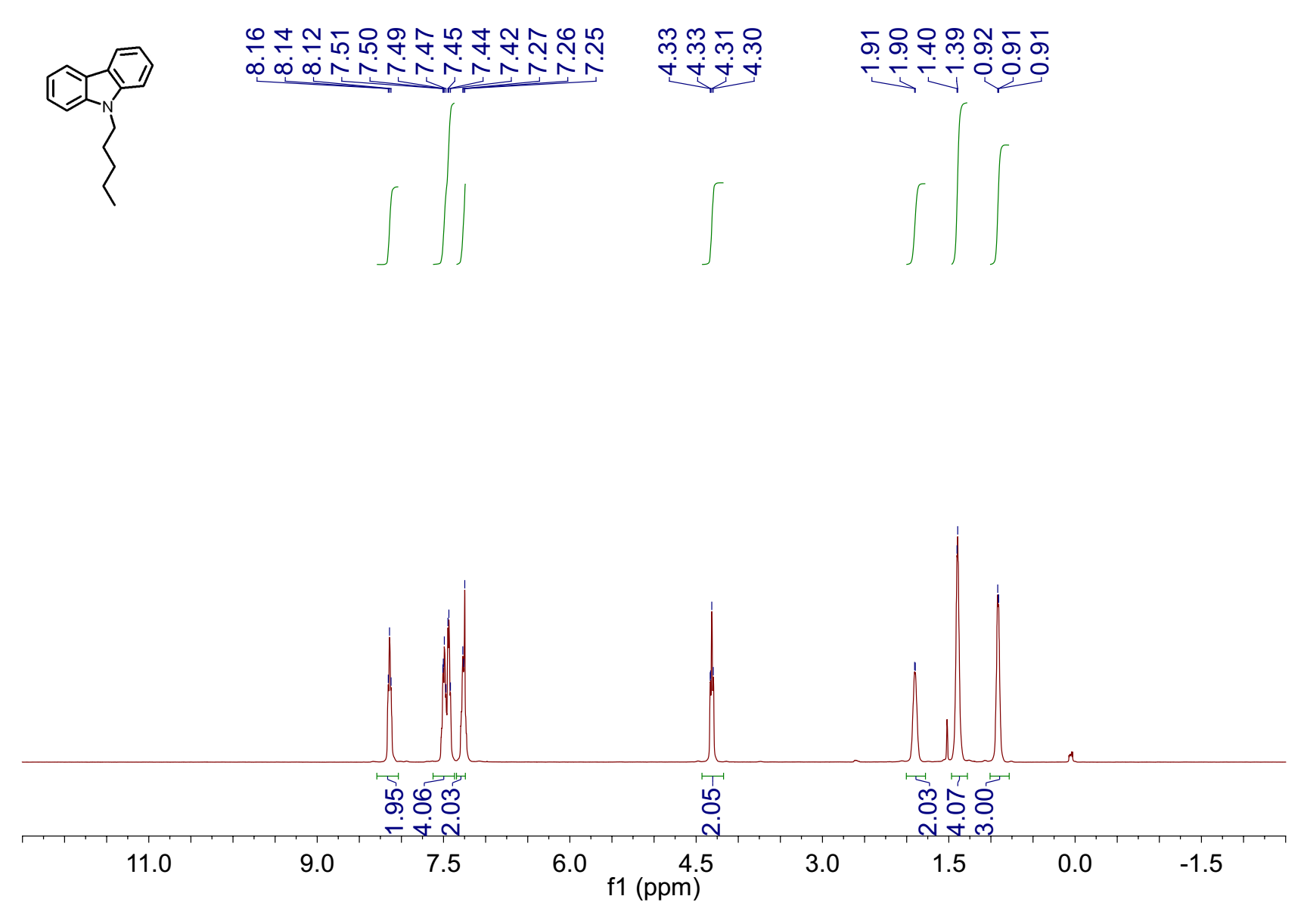


**Figure S26.** ^1^H NMR spectrum of compound 5 in CDCl_3_.


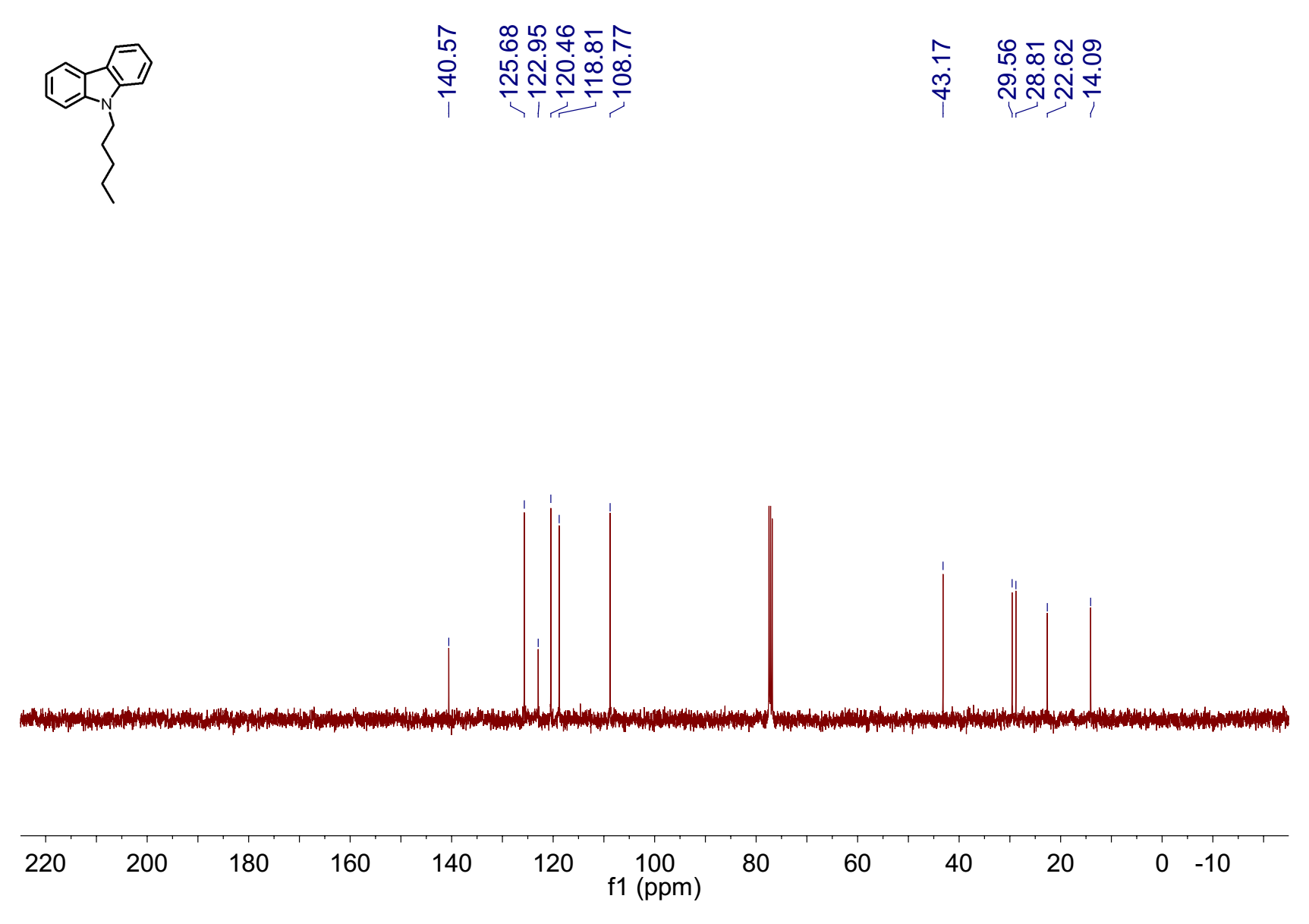


**Figure S27.** ^13^C NMR spectrum of compound 5 in CDCl_3_.


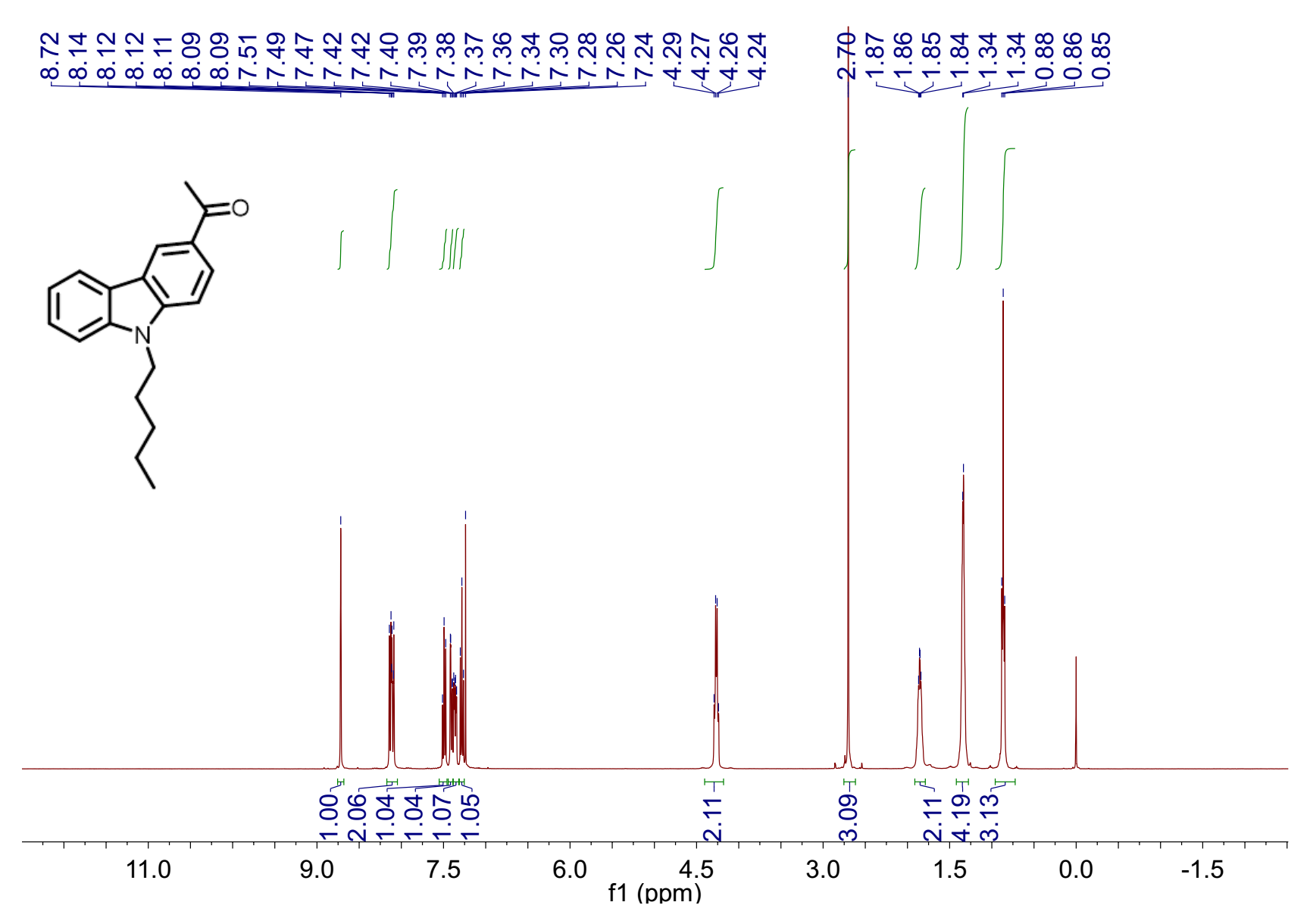


**Figure S28.** ^1^H NMR spectrum of compound 6 in CDCl_3_.


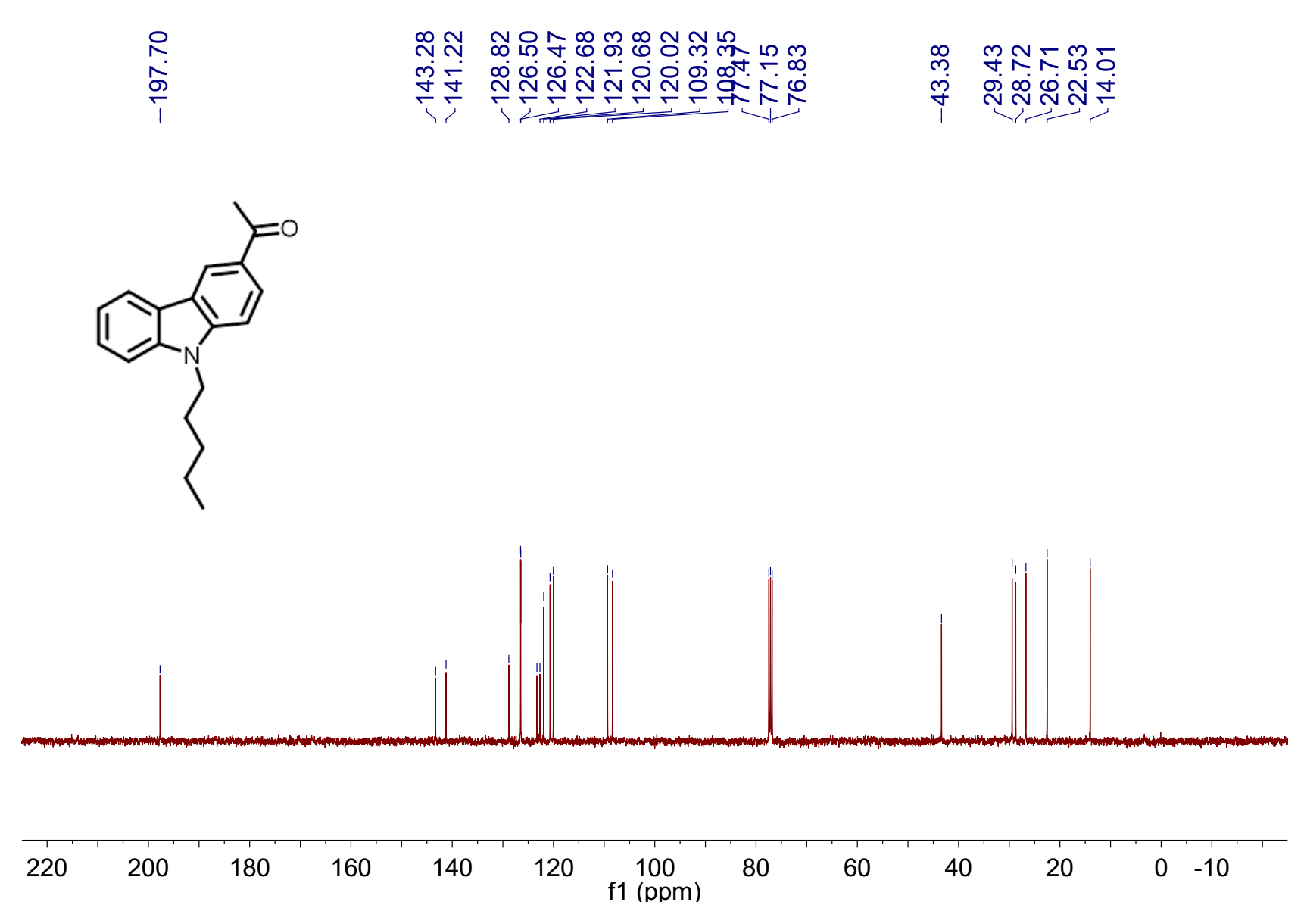


**Figure S29.** ^13^C NMR spectrum of compound 6 in CDCl_3_.


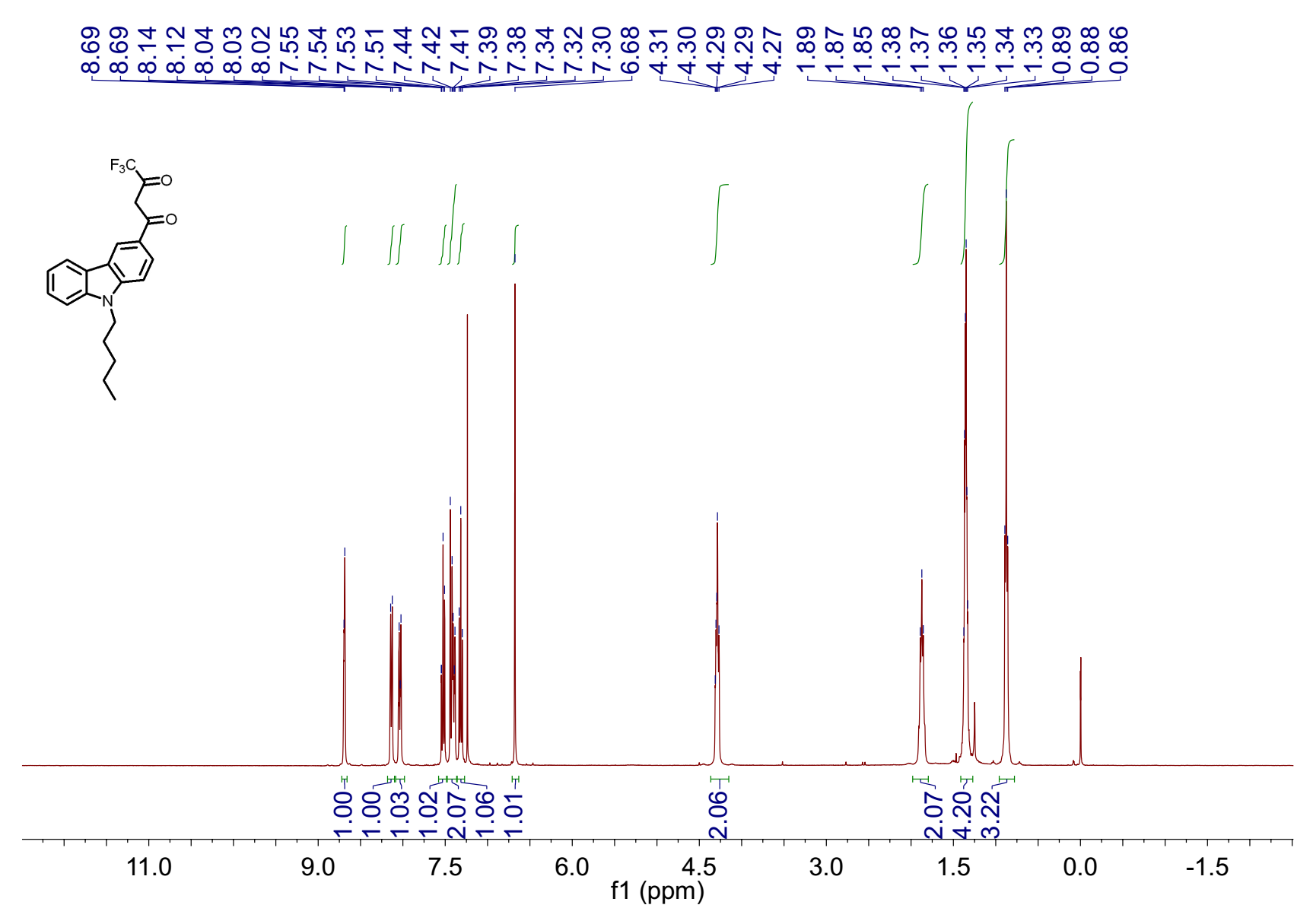


**Figure S30.** ^1^H NMR spectrum of compound 7 in CDCl_3_.


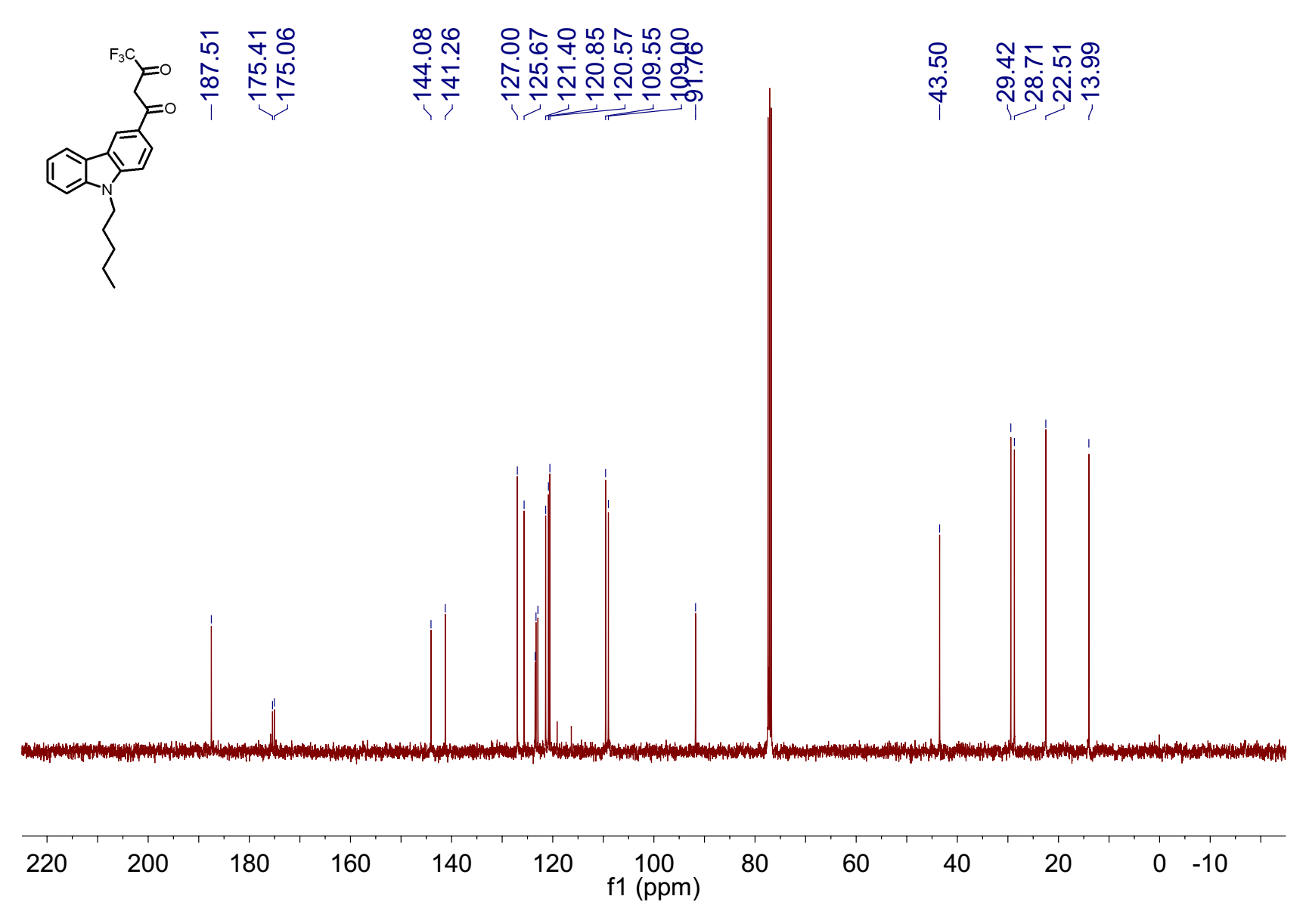


**Figure S31.** ^13^C NMR spectrum of compound 7 in CDCl_3_.


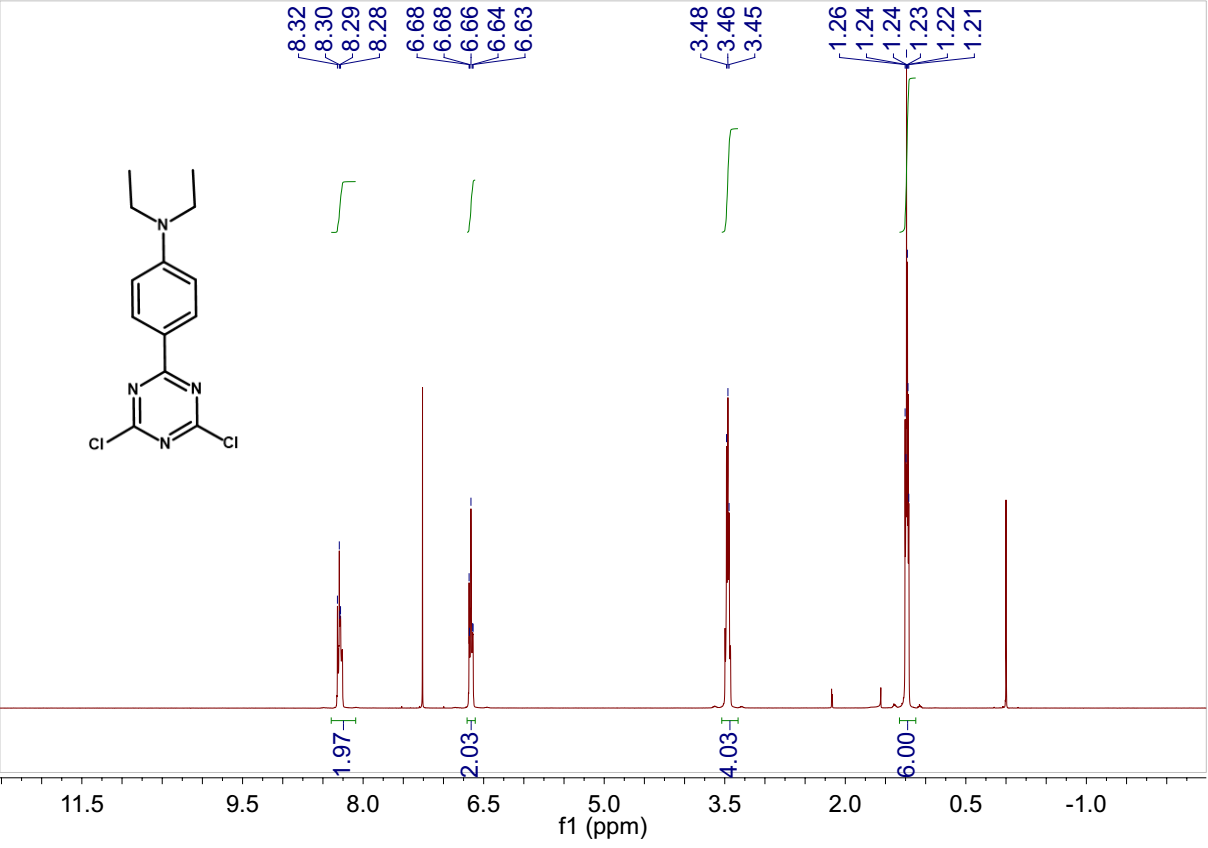


**Figure S32.** ^1^H NMR spectrum of compound 9 in CDCl_3_.


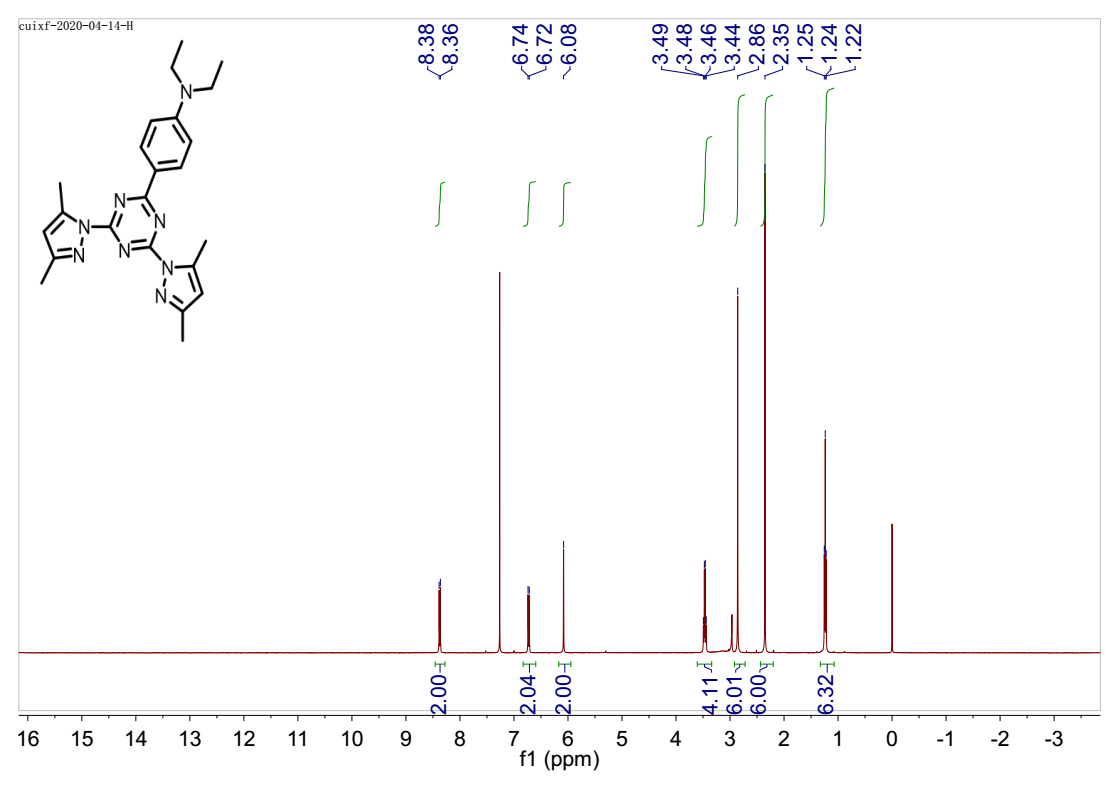


**Figure S33.** ^1^H NMR spectrum of compound 10 in CDCl_3_.


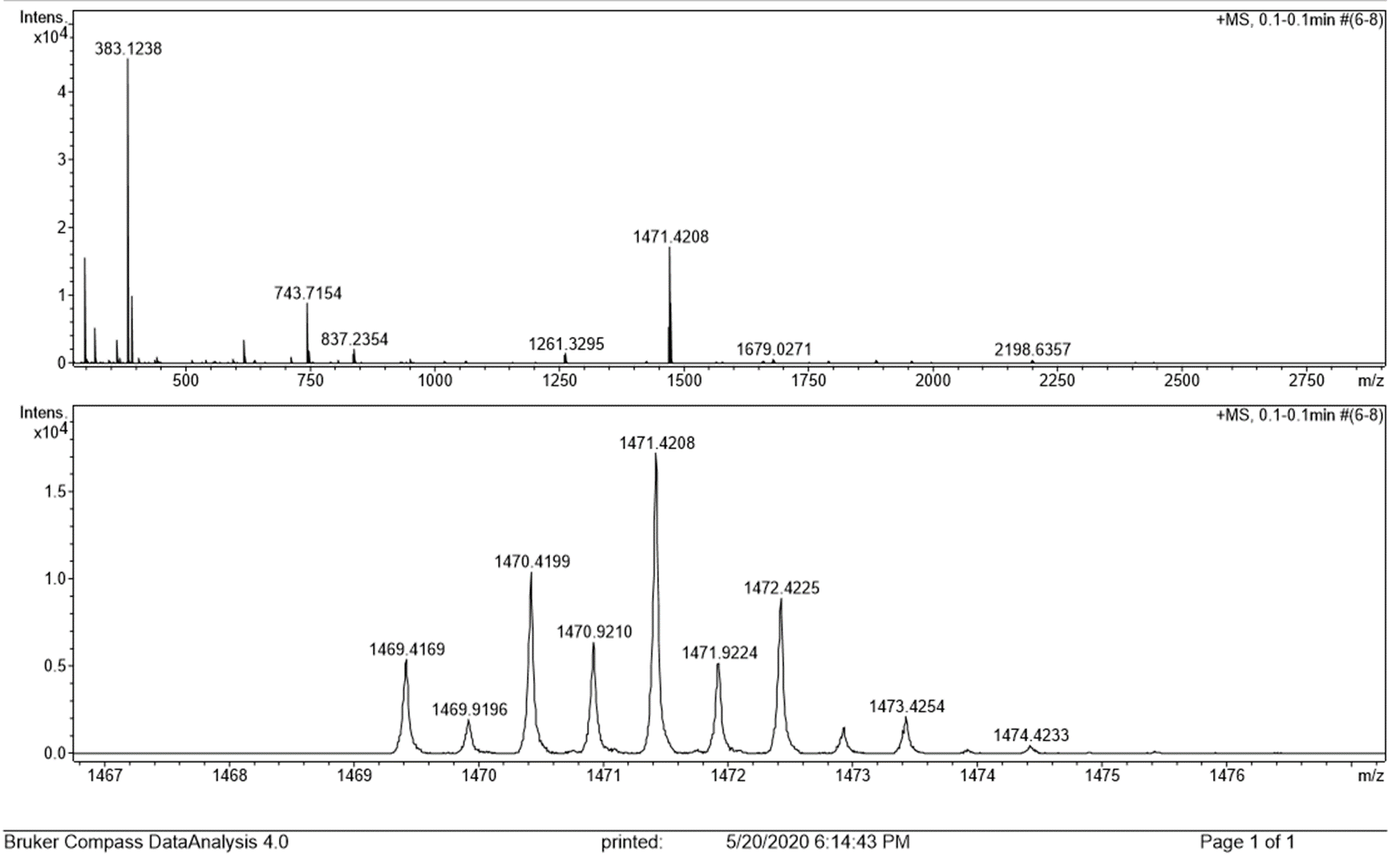


**Figure S34.** HRMS spectrum of the Eu^3+^ complex.


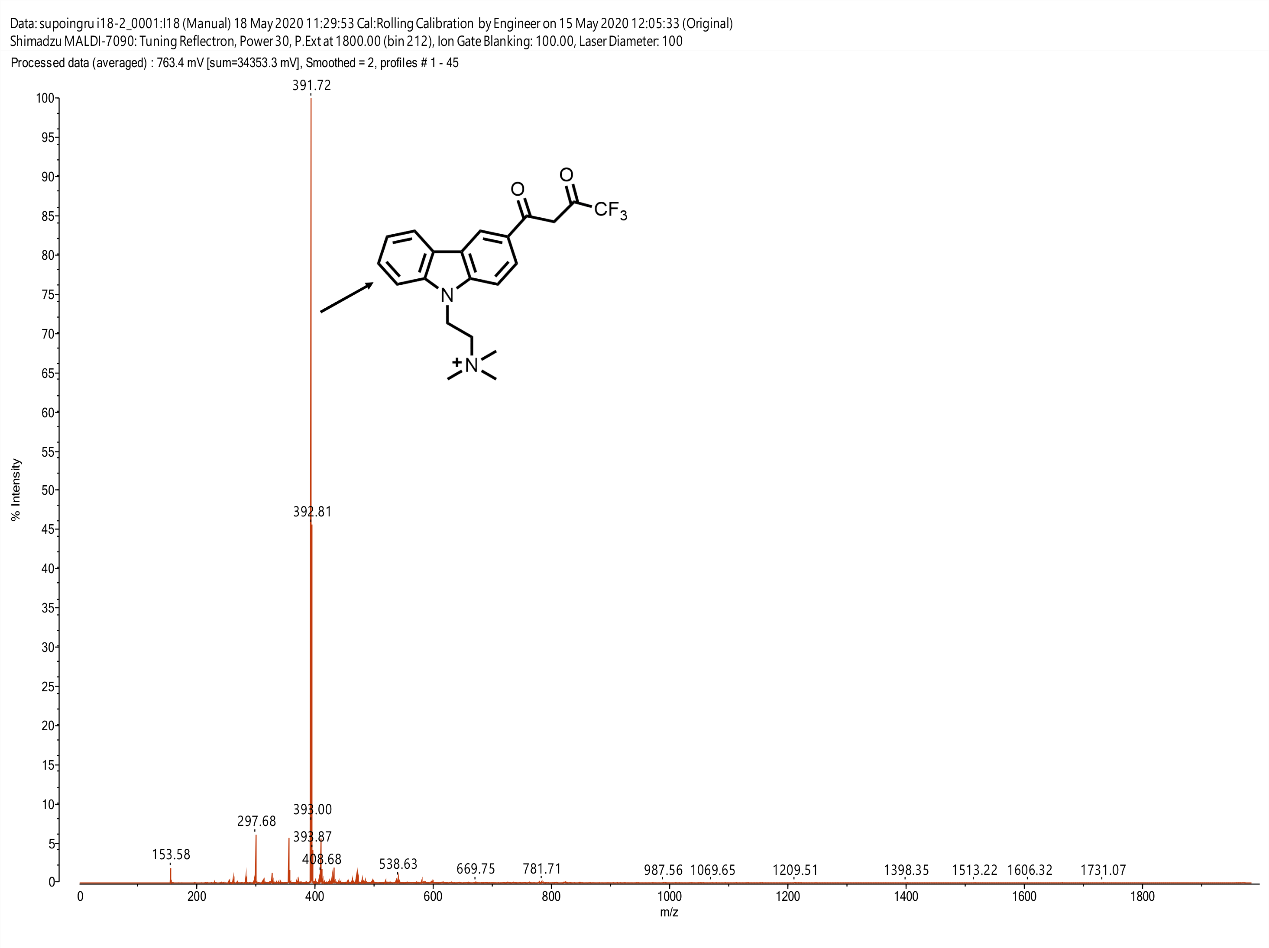


**Figure S35.** MALDI-TOF mass spectrum of compound 4.


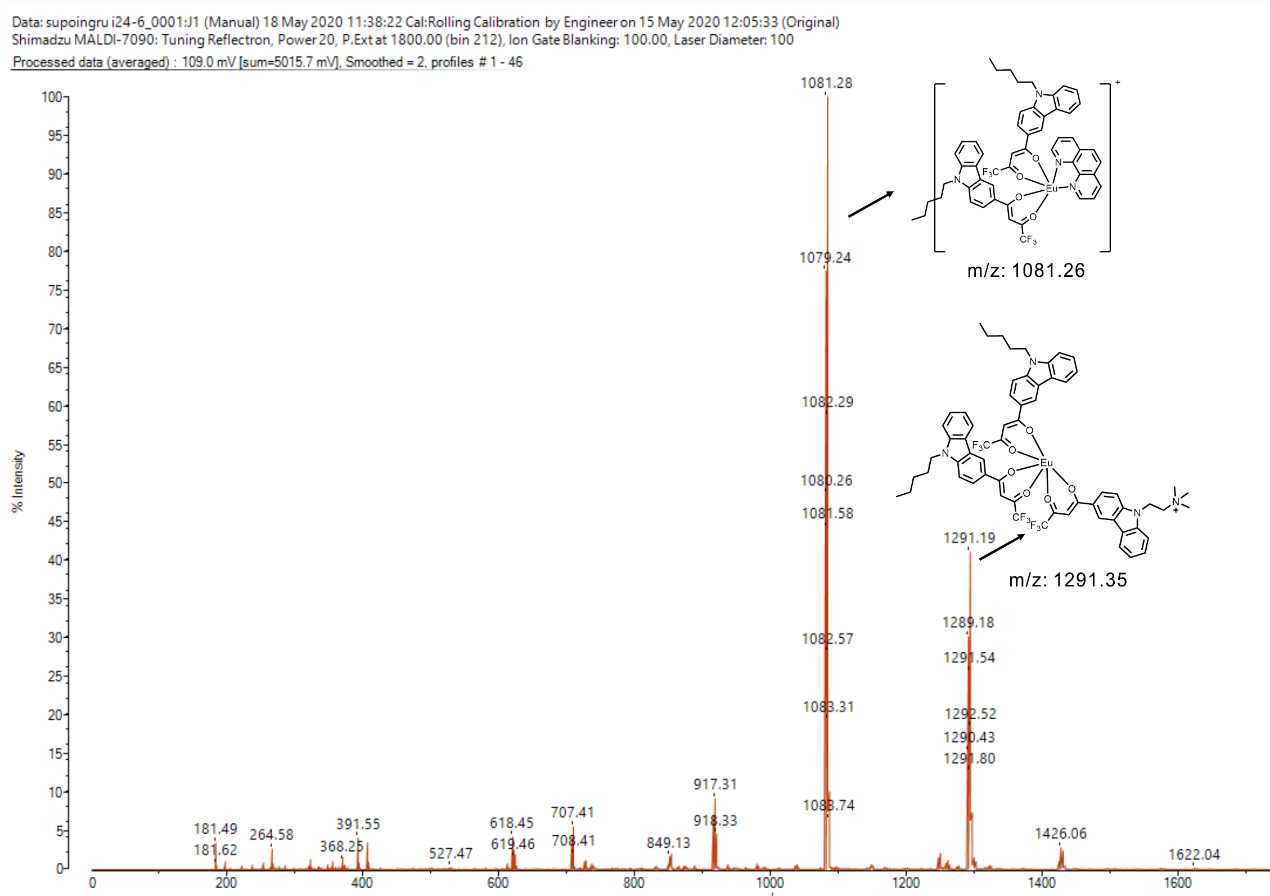


**Figure S36.** MALDI-TOF mass spectrum of Eu^3+^ complex.


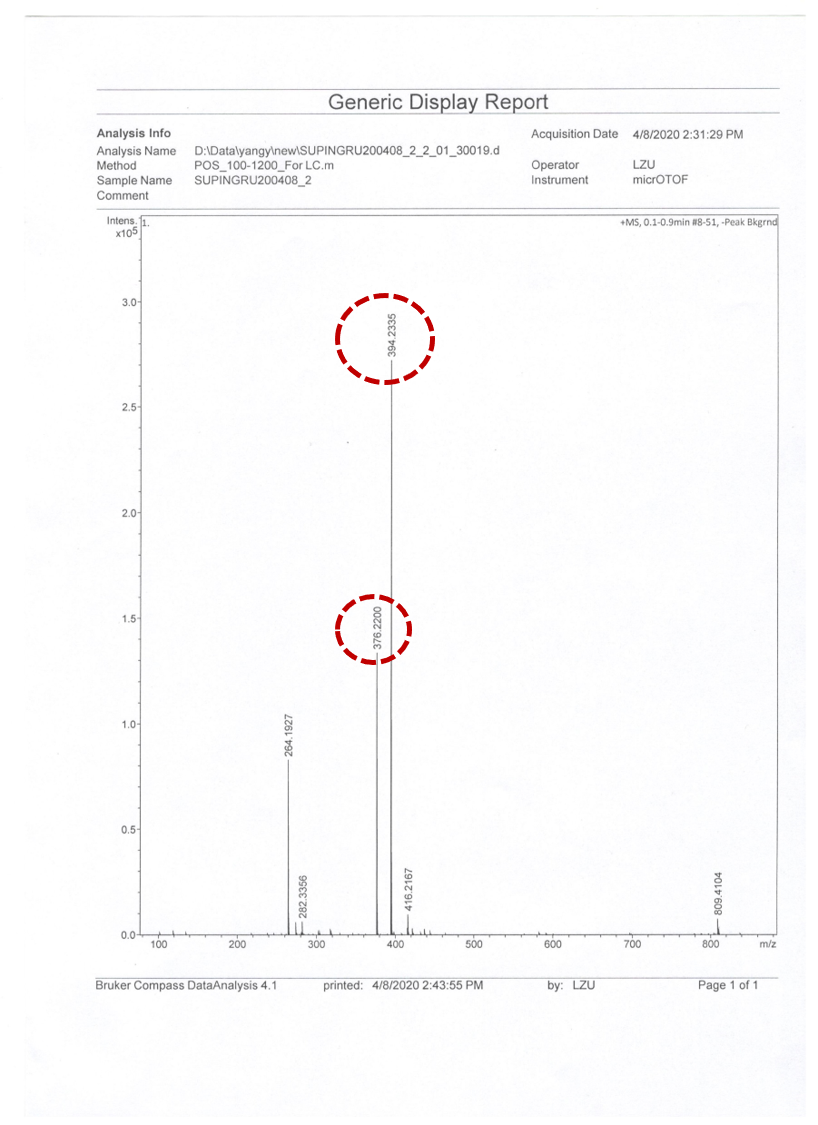


**Figure S37.** ESI**-**MS spectra of the compound 7.

**3. Supporting Tables**

**Table S1.** Luminescence quantum yield and lifetime of Eu^3+^ complexes (50 μM) in different acetone/water mixtures.

| H_2_O/D_2_O Fractions (%) | 0 | 10 | 20 | 30 | 40 | 50 | 60 | 70 | 80 | 90 |
| --- | --- | --- | --- | --- | --- | --- | --- | --- | --- | --- |
| Lifetime  (μs)  (in H_2_O) | 673.7±0.1 | 445.3±0.1 | 400.9±0.1 | 398.0±0.1 | 470.3±0.1 | 517.5±0.1 | 534.8±0.1 | 710.2±0.1 | 728.5±0.1 | 755.4±0.1 |
| Lifetime  (μs)  (in D_2_O) |  | 563.5±0.1 | 563.8±0.1 | 564.4±0.1 | 572.8±0.1 | 559.8±0.1 | 572.0±0.1 | 596.1±0.1 | 760.5±0.1 | 747.1±0.1 |
| q |  | 0.3±0.1 | 0.6±0.1 | 0.6±0.1 | 0.2±0.1 | -0.1±0.1 | -0.2±0.1 | -0.4±0.1 | -0.2±0.1 | -0.3±0.1 |
| *Ф*_overall_ (%) | 18.7±0.1 | 9.2±0.1 | 6.9±0.1 | 5.1±0.1 | 3.4±0.1 | 11.6±0.1 | 24.0±0.1 | 15.9±0.1 | 14.8±0.1 | 10.8±0.1 |
| *Ф*_sen_ (%) | 15.6±0.1 | 7.7±0.1 | 5.8±0.1 | 4.3±0.1 | 2.8±0.1 | 9.6±0.1 | 20.0±0.1 | 13.3±0.1 | 12.4±0.1 | 9.0±0.1 |

**Table S2.** Sensitivity and temperature operation range of Ln^3+^-based luminescent thermometers in recently.

| Thermometers | Sensitivity  (% intensity/K) | Temperature range/℃ | Temperature dependence  algorithm | References |
| --- | --- | --- | --- | --- |
| Eu β-diketonate  complex | 5.6-2.0 | 60-10 | ^5^D_0_ - ^7^F_2_ intensity | *J. Alloys Compd.,* 2008, 453, L1–L3 |
| Eu hybrid Nanoparticles | ~3.0 or ~2.0 | 45-25 | ^5^D_0_ - ^7^F_2_ intensity and  ^5^D_0_ lifetime | *Adv. Mater., 2010, 22, 716–719* |
| Eu MOF | 0.6-0.48  and 0.23-0.21 | 60-20 and 65-25 | ^5^D_0_ - ^7^F_2_ intensity and  ^5^D_0_ lifetime | *Anal. Chem. 2019, 91, 5225−5234* |
| Upconversion  Nanocomposite | ~1 | 40-35 | ^2^H_11/2_ - ^4^I_15/2_  and ^4^S_3/2_ - ^4^I_15/2_ intensity | *Nature Communications* 2016, 7, 10 |
| Tb/Eu-MOF | 0.34-0.59 | 150-30 | ^5^D_4_ - ^7^F_5_ and ^5^D_0_ - ^7^F_2_ intensity | *Inorg. Chem. 2018, 57, 5, 2620–2630* |
| Upconversion  Nanocomposite | ~1 | 40-35 | ^2^H_11/2_ - ^4^I_15/2_  and ^4^S_3/2_ - ^4^I_15/2_ intensity | *Nature Communications* 2016, 7, 10 |
| La_0.999_Nd_0.001_PO_4_ | 1.6-0.2 | 27-500 | ^4^F_5/2_ – ^4^F_3/2_ gap for Boltzmann-based luminescence | *Nanomaterials 2020, 10, 543* |
| Eu^3+^ NPs | **5.1-2.3**  **and**  **5.3-4.8** | **55-25**  **and**  **45-25** | **^5^D_0_ - ^7^F_2_ intensity and**  **^5^D_0_ lifetime** | **This work** |

**4. REFERENCES**

1. Zhang Y, Shen TT and Kirillov AM et al. NIR light/H_2_O_2_-triggered nanocomposites for a highly efficient and selective synergistic photodynamic and photothermal therapy against hypoxic tumor cells. *Chem Commun* 2016; **52**: 7939-42.

2. Lou ZR, Li P and Song*,* et al. Ratiometric fluorescence imaging of cellular hypochlorous acid based on heptamethine cyanine dyes. *Analyst* 2013; **138**(21): 6291-5.

3. Purushothaman B, Arumugam, P, Ju, H, et al. Novel ruthenium(II) triazine complex Ru(bdpta)(tpy) (2+) co-targeting drug resistant GRP78 and subcellular organelles in cancer stem cells. *Eur J Med Chem* 2018; **156**: 747-59.

4. Yang C, Fu LM and Wang Y et al. A highly luminescent europium complex showing visible-light-sensitized red emission: Direct observation of the singlet pathway. *Angew Chem Int Ed* 2004; **43**(38): 5010-3.

5. Hu ZJ, Tian XH and Zhao XH et al. Efficient two-photon-sensitized luminescence of a novel europium(III) beta-diketonate complex and application in biological imaging. *Chem Commun* 2011; **47**(46): 12467-9.
